# PhoP-regulated VirK acts as an accessory factor to maintain virulence in polymyxin-resistant *Klebsiella pneumoniae*

**DOI:** 10.1093/nar/gkag290

**Published:** 2026-04-25

**Authors:** Haibin Li, Longyang Jin, Penghe Wang, Ruobing Wang, Qi Wang, Xiukun Wang, Congran Li, Xuefu You, Hui Wang

**Affiliations:** Department of Clinical Laboratory, Peking University People’s Hospital, Beijing, 100044, China; Department of Clinical Laboratory, Peking University People’s Hospital, Beijing, 100044, China; Beijing Key Laboratory of Technology and Application for Anti-infective New Drugs Research and Development, Institute of Medicinal Biotechnology, Chinese Academy of Medical Sciences & Peking Union Medical College, Beijing, 100050, China; Department of Clinical Laboratory, Peking University People’s Hospital, Beijing, 100044, China; Department of Clinical Laboratory, Peking University People’s Hospital, Beijing, 100044, China; Beijing Key Laboratory of Technology and Application for Anti-infective New Drugs Research and Development, Institute of Medicinal Biotechnology, Chinese Academy of Medical Sciences & Peking Union Medical College, Beijing, 100050, China; Beijing Key Laboratory of Technology and Application for Anti-infective New Drugs Research and Development, Institute of Medicinal Biotechnology, Chinese Academy of Medical Sciences & Peking Union Medical College, Beijing, 100050, China; Beijing Key Laboratory of Technology and Application for Anti-infective New Drugs Research and Development, Institute of Medicinal Biotechnology, Chinese Academy of Medical Sciences & Peking Union Medical College, Beijing, 100050, China; State Key Laboratory of Bioactive Substances and Functions of Natural Medicines, Institute of Medicinal Biotechnology, Chinese Academy of Medical Sciences & Peking Union Medical College, Beijing, 100050, China; Department of Clinical Laboratory, Peking University People’s Hospital, Beijing, 100044, China; Institute of Medical Technology, Peking University Health Science Center, Beijing, 100191, China

## Abstract

The genetic connection between virulence and antibiotic resistance remains poorly understood. Our previous RNA-Seq analysis of a polymyxin-resistant *Klebsiella pneumoniae* ATCC BAA2146 mutant identified a highly expressed VirK/YbjX family gene (kpn2146_RS17285), encoding a conserved membrane protein, designated *virK* (**vi**rulence **r**equired for ***K**lebsiella pneumoniae*). While PhoP-dependent antibiotic resistance is mediated through established pathways such as *arn/pmr*, we identify VirK as a PhoP-regulated factor specifically contributing to virulence. VirK localizes to the outer membrane and, although not involved in lipopolysaccharide modification, its deletion modestly reduced bacterial virulence in a mouse systemic infection model. Transcriptional and electrophoretic mobility shift assays demonstrated that *virK* is directly activated by the PhoP protein. A strong positive correlation between *virK* and *phoP* expression (*r* = 0.98) was also observed in multidrug-resistant clinical isolates. Since the PhoP/PhoQ two-component system mediates polymyxin resistance, its direct regulation of VirK uncovers an adaptive mechanism coupling enhanced virulence with antibiotic resistance. These findings reveal a previously unrecognized PhoP/VirK regulatory pathway that contributes to pathogenicity in polymyxin-resistant *Klebsiella pneumoniae*, offering new insights into bacterial evolution and suggesting that targeting PhoP/PhoQ could provide an effective strategy to combat multidrug-resistant *K. pneumoniae* infections.

## Graphical Abstract

**Graphical Abstract:**
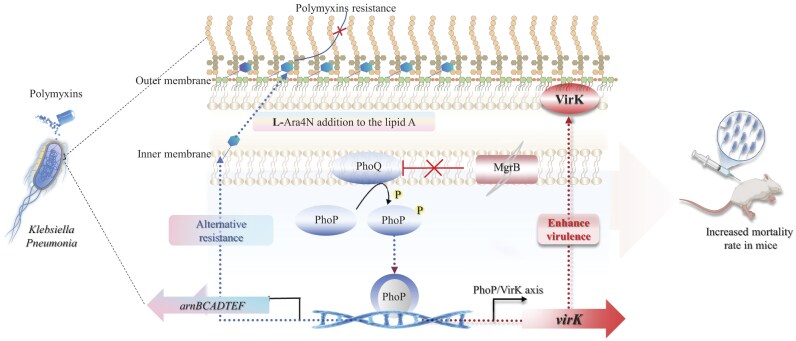

## Introduction

In *Klebsiella pneumoniae*, the histidine kinase PhoQ senses stress conditions, activating two-component systems (PhoP/PhoQ) and subsequently inducing the *arnBCADTEF* operons to synthesize and incorporate 4-amino-4-deoxy-l-arabinose (L-Ara4N) into lipid A [1, 2]. Overexpression of the *phoP/phoQ* operon can be induced by inactivation of MgrB (a negative regulator of the PhoP/PhoQ system), which decreases the susceptibility of the strain to polymyxins. However, the effects of the *mgrB* mutation on *K. pneumoniae* virulence are controversial, with increased *K. pneumoniae* virulence [3, 4] or no modification in virulence [5] reported. Specific *mgrB* mutations in individual strains resulted in either loss of virulence or no effect/increased virulence. These conflicting results suggest possible unidentified mechanisms for polymyxin virulence in the *mgrB* mutations.

VirK/YbjX proteins are unknown functional proteins containing the DUF535 domain, and VirK protein homologs in the UniProt database are annotated as virulence factors, antimicrobial peptide resistance proteins, YbjX proteins, and unidentified proteins. Members of the VirK/YbjX (DUF535) family are present in many pathogenic bacteria. Some indirect evidence suggests that the *virK* gene is functionally related to or associated with LPS modification and may regulate its activity by localizing other bacterial proteins to appropriate positions or maintaining correct interactions with virulence-related proteins such as lipid A [6, 7]. The *virK* gene has been reported to be necessary for the localization of the self-transporter protein IcsA to the bacterial surface and subsequent intracellular transmission in *Shigella* [8]. In addition, the *virK* gene may be involved in maintaining the virulence of pathogenic bacteria, and it has been confirmed in *Salmonella* that the *virK* gene contributes to the reshaping of the host environment via the bacterial outer membrane [9]. The outer membrane protein YbjX, also belonging to the VirK/YbjX (DUF535) family, is an enzyme responsible for adding a portion of myristic acid to lipid A. Mutations in the YbjX gene may weaken the pathogenicity of avian pathogenic *E. coli* (APEC), not only by affecting bacterial adhesion and invasion but also by increasing bacterial outer membrane permeability to reduce resistance to avian beta-defense factors.

During preliminary investigations of a polymyxin-resistant mutant (Mut-S) of *K. pneumoniae* ATCC BAA-2146 (Kpn2146) [10], we detected highly upregulated expression of the Kpn2146:RS17285 gene. The protein amino acid sequence encoded by this gene is significantly similar to that of the virulence genes of the VirK/YbjX (DUF535) family, and the open reading frame Kpn2146:RS17285 is referred to as the *virK* gene. Compared with the parental strain, Mut-S increased polymyxin resistance (32–64 times greater) caused by the *mgrB* mutation and the subsequent upregulation of the *phoP/phoQ* two-component system (TCS). Further identification of the highly upregulated expression of the possible virulence-related gene *virK* encouraged us to explore the function and regulatory network of the *virK* gene in *K. pneumoniae* and to examine the possible synergistic regulatory effects of the PhoP/PhoQ on polymyxin resistance and virulence.

## Materials and methods

### Bacterial strains, culture media, plasmids, and primers

The bacterial strains and plasmids used in this study have been listed in Supplementary Table S1-S5. Genetic background information for the clinical isolates, including mutations in *phoPQ, mgrB*, and other regulatory loci, was obtained from historical sequencing and clinical records where available (Supplementary Table S6). *K. pneumoniae* strains were cultured in Luria-Bertani (LB) broth (Sigma-Aldrich) at 37°C with shaking at 200 rpm or on LB agar plates, unless otherwise specified. In both experiments and genetic tool editing, antibiotics were supplemented at standard concentrations: 50 mg/L of kanamycin, 50 mg/L of apramycin, and 50 mg/L of spectinomycin. Antibiotics were purchased from MedChemExpress (MCE). All strains were stored in 20% glycerol at -80°C.

### Virulence of the strains in the mouse systemic infection model

Strains were subjected to *in vivo* virulence comparison using a murine systemic infection model by intraperitoneal injection in the presence of 5% mucin. CD-1 (ICR) mice (19–21 g, half male, half female) were purchased from Vital River Laboratories (Beijing, China). All animals were housed under controlled humidity (30%∼70%), temperature (22 ± 3°C), and a 12 h light-dark cycle. Cell pellets from fresh overnight cell cultures were properly diluted in saline and then further diluted 10-fold in 5% mucin. Five infection doses were used for each strain. 0.5 mL of the bacterial suspension in 5% mucin was injected intraperitoneally into each mouse randomly allocated to different groups. During the virulence testing, mice were monitored twice daily for clinical signs of infection or distress, including body weight, appearance/posture, activity/responsiveness, and respiratory condition. Animals were humanely euthanized when the body weight decreased to ≤ 13 g, or when severe respiratory distress/moribund state was observed. Euthanasia was performed using carbon dioxide (CO₂) in accordance with institutional guidelines. Animal deaths were recorded for 7 days post-infection, and the median lethal doses needed to kill 50% of the mice (LD_50_) and 99% of the mice (LD_99_), and the corresponding 95% confidence intervals (CI) were calculated by the Probit method. The blood samples were harvested at 2 and 8 h after infection, and the sera were obtained by centrifugation after the blood samples were left at 25°C for 30 min. Serum biochemical indices were measured using a Fully Automated Biochemistry Analyzer (Hitachi Diagnostic Products Corporation, Japan).

### Gene deletion using the CRISPR-Cas9 system

The *phoP* gene deletion mutant in the polymyxin S_2_-resistant Kpn2146 (Mut-S) strain was successfully obtained using the pCasKP-pSGKP genome editing system according to methods described previously [11]. The wild-type Kpn2146 was first transformed with the pCasKP-apr plasmid via electroporation, using parameters of 2500 V, 200 Ω, 25 μF in a 2-mm cuvette. Subsequently, after induction with L-arabinose, the cells containing the pCasKP-apr plasmid were prepared as competent cells. The annealed *phoP* spacer (designed by CRISPOR [12]) oligonucleotides were inserted into the *Bsa*I sites of the pSGKP-spe plasmid by Golden Gate assembly. The *phoP* spacer-introduced pSGKP-spe plasmid and the corresponding 90-nucleotide (nt) single-strand DNA (ssDNA) repair template were then co-electroporated into the aforementioned pCasKP-apr-harboring competent cells. The colonies were selected on an LB agar plate containing apramycin (50 μg/mL) and spectinomycin (1 mg/mL) at 30°C. The successful deletion of the *phoP* gene in the Mut-S, designated as Mut-S-Δ*phoP*, was confirmed through genetic and phenotypic analyses by PCR and sequencing. After obtaining the *phoP* deletion mutant, both the pCasKP-apr and the *phoP* spacer-introduced pSGKP-spe plasmids were cured by culturing the cells at 37°C with 5% (wt/vol) sucrose. The Kpn2146Δ*virK* and Mut-SΔ*virK* strains were created using the same method.

### Complementation assays

To determine whether the complete deletion of the *phoP* gene was affecting *virK* gene expression, a complementation assay of *phoP* was performed. The full-length *phoP* gene from colistin-susceptible Kpn2146 was amplified by PCR using the primers *phoP*-C-F and *phoP*-C-R (Supplementary Table S1) and ligated into a fragment amplified from pTOPO-*mgrB* (Apr^r^) by primers pTOPO-P-F and pTOPO-P-R (Supplementary Table S1). The assembly was conducted using Gibson Assembly Master Mix (Cat. E2611S, NEB, Gene Company, China) according to the manufacturer’s instructions, and transformed into electrocompetent *E. coli* top10 strains (Tsingke Biotechnology, China) by electroporation. Transformants were selected by overnight incubation at 37°C on Mueller–Hinton agar supplemented with apramycin (50 mg/L, Cat.HY-B1329, MedChemExpress, China). Plasmids were isolated using the RapidLyse Plasmid Mini Kit (Cat. DC211-02, Vazyme, China), confirmed by KBSeq (Sangon, China), and the correct pTOPO-*phoP* (Apr^r^) plasmids were then introduced into Mut-S, respectively, by electroporation. The transformants were selected on plates containing apramycin at a concentration of 50 mg/L. The pTOPO-flag-*phoP* (Apr^r^) plasmid was constructed for validation of PhoP regulation of *virK* gene expression through a ChIP-seq experiment. The full-length *phoP* gene from colistin-susceptible Kpn2146 was amplified by PCR using the primers *phoP*-F-F and *phoP*-F-R (Supplementary Table S1) and ligated into a fragment amplified from pTOPO-*mgrB* (Apr^r^) by primers pTOPO-P-F-F and pTOPO-P-F-R (Supplementary Table S1). The correct pTOPO-flag-*phoP* (Apr^r^) plasmids verified by sequencing were then introduced into Kpn2146 by electroporation.

To further confirm the function of the *virK* gene, a complementation assay was performed for *virK* mutants. The full-length *virK* gene from colistin-susceptible Kpn2146 was amplified by PCR using the primers *virK*-C-F and *virK*-C-R (Supplementary Table S1) and ligated into a fragment amplified from pTOPO-*mgrB* (Apr^r^) by primers pEASY-V-F and pEASY-V-R (Supplementary Table S1). The transformants containing pEASY-*virK* (Apr^r^) were selected on plates containing 50 mg/L apramycin. The subcellular localization study of VirK protein was achieved using the Kpn2146-Δ*virK* strain supplemented with pEASY-*virK-his6* (Apr^r^). The *virK* gene was first cloned into plasmid pET28a using 5′ NcoI and 3′ XhoI restriction enzymes, resulting in a pET28a plasmid carrying C-terminal 6 × His tagged *virK* (pET28a-virK-his6). The pET28a-*virK*-His6 plasmid was verified through sequencing by TSINGKE Corporation (Beijing, China). The full-length *virK*-His6 region derived from the pET28a-*virK*-his6 plasmid was then amplified by PCR using primers *virK*-C-F and *virK*-C-R (Supplementary Table S1) and subsequently ligated into a fragment amplified from pTOPO-*mgrB* (Apr^r^) using primers pEASY-V-H-F and pEASY-V-H-R (Supplementary Table S1) for constructing pEASY-*virK-his6* (Apr^r^) with similar procedures as for constructing pTOPO-*phoP* (Apr^r^). The correct pTOPO-*virK* (Apr^r^) and pEASY-*virK-his6* (Apr^r^) plasmids were verified by sequencing and introduced into Kpn2146-Δ*virK*, respectively, by electroporation.

### Real-time quantitative PCR

The extraction method of total RNA was the same as that of transcriptome sequencing. The RNA levels of *mgrB, phoP, virK*, and *23S rRNA* (serving as an internal control) were quantified using the HiScript II One Step qRT-PCR SYBR Green Kit (Catalog No. Q221, Vazyme Biotech, China) on a qTOWER^3^ Real-Time PCR (qPCR) System (Analytik Jena), following the manufacturer’s instructions. All specific primers (Supplementary Table S1) were designed according to the genome of Kpn2146 (NCBI reference sequence: NZ_CP006659.2). The relative RNA expression levels were calculated according to the 2^−△△Ct^ method with normalization to the 23S rRNA levels. RT-qPCR was performed by three biological repeats with three technical replications.

### ChIP-seq analysis

Chromatin immunoprecipitation assays were performed according to procedures previously described [13] by Wuhan IGENEBOOK Biotechnology Co., Ltd. Briefly, Kpn2146-Flag-*phoP* cells were washed twice in cold PBS buffer and cross-linked with 1% formaldehyde for 10 min at room temperature and then quenched by the addition of glycine (125 mmol/L final concentration). Afterwards, samples were lysed with 50 mM Tris–HCl (pH 8.0), 10 mM EDTA, 1% SDS, 1 × protease inhibitor cocktail, and chromatins were obtained on ice. Chromatins were sonicated to get soluble sheared chromatin (average DNA length of 200–500 bp). 20 μL chromatin was stored at -20°C as input DNA, and 100 μL chromatin was used for immunoprecipitation by HRP-conjugated anti-Flag antibodies. 10 μg of antibody was used in the immunoprecipitation reactions at 4°C overnight. The next day, 30 μL of protein beads was added, and the samples were further incubated for 3 h. The beads were then washed once with 20 mM Tris–HCl (pH 8.1), 50 mM NaCl, 2 mM EDTA, 1% Triton X-100, 0.1% SDS; twice with 10 mM Tris–HCl (pH 8.1), 250 mM LiCl, 1 mM EDTA, 1% NP-40, 1% deoxycholic acid; and twice with TE buffer (10 mM Tris–HCl at pH 7.5, 1 mM EDTA). Bound material was then eluted from the beads with 300 μL of elution buffer (100 mM NaHCO_3_, 1% SDS), treated first with RNase A (final concentration 8 μg/mL) for 6 h at 65°C and then with proteinase K (final concentration 345 μg/mL) overnight at 45°C. Immunoprecipitated DNA was used to construct sequencing libraries following the protocol provided by the I NEXTFLEX^®^ ChIP-Seq Library Prep Kit for Illumina^®^ Sequencing (Cat. NOVA-5143–02, Bioo Scientific) and sequenced on Illumina NovaSeq 6000 with PE 150 method.

### Data analysis

Trimmomatic (version 0.36) was used to filter out low-quality reads [2]. Clean reads were mapped to the *K. pneumoniae* (ATCC® BAA-*2146*™) genome by Bwa (version 0.7.15) [3]. SAMtools (version 1.3.1) was used to remove potential PCR duplicates [4]. MACS2 software (version 2.1.1.20160309) was used to call peaks by default parameters (bandwidth, 300 bp; model fold, 5, 50; q value, 0.05). If the midpoint of a peak was located closest to the TSS of one gene, the peak was assigned to that gene [5]. HOMER (version 3) was used to predict motif occurrence within peaks with default settings for a maximum motif length of 12 base pairs [6]. ClusterProfiler (http://www.bioconductor.org/packages/release/bioc/html/clusterProfiler.html) in the R package [7] was employed to perform GO [8] (Gene Ontology, http://geneontology.org/) and KEGG [9] (Kyoto Encyclopedia of Genes and Genomes, http://www.genome.jp/kegg/) enrichment analysis. The GO and KEGG enrichment analyses were calculated using a hypergeometric distribution with a q-value cutoff of 0.05.

### ChIP-qPCR

ChIP experiments were performed as described above. The enrichment of DNA fragments bound by YrpA, GreB, and Crp was quantified by quantitative PCR (qPCR) using Power SYBR Green PCR Master Mix on an Applied Biosystems QuantStudio^TM^ 5 Real-Time PCR system. The relative enrichment of target DNA regions was calculated using the 2^−ΔΔCt^ method.

### Cloning and recombinant protein purification

The *phoP* gene was cloned into the plasmid pET28a using 5′ *Nde*I and 3′ *Xho*I restriction enzymes, resulting in a *N*-terminal 6 × His-tagged-*phoP* plasmid (pET28a-*phoP*-his6). The pET28a-*phoP*-his6 plasmid was verified through sequencing by GENEWIZ Corporation (Suzhou, China) and transferred into *E. coli* strain BL21 (DE3). For PhoP purification, the *E. coli* strain BL21 (DE3) carrying pET28a-*phoP*-his6 was grown in LB medium to an OD_600_ of 0.6, followed by induction with 0.2 mM isopropyl β-D-thiogalactoside (IPTG) for 5 h at 18°C with shaking at 160 rpm. The bacterial cells were harvested by centrifugation at 3500 × *g* (20 min, 4°C) and resuspended in a cold lysis buffer (50 mM Na_2_HPO_4_, 50 mM NaH_2_PO_4_, 300 mM NaCl). After sonication on ice (3 s on, 3 s off, for 5 min), the mixture was subjected to centrifugation at 10 000 × *g* for 10 min at 4°C. The supernatant was collected and mixed with the PureCube Ni-NTA Agarose (Cube Biotech, Germany) and incubated at 4°C for 2 h with gentle shaking. The suspensions were washed three times with the lysis buffer containing 20 mM imidazole, and the bound protein was then eluted with the lysis buffer containing 300 mM imidazole. The purified recombinant protein was desalinated using Amicon^®^ Ultra Centrifugal Filter (Cat. UFC9010, Sigma, USA), and the quality of the purified PhoP was assessed using SDS-PAGE and Coomassie Brilliant Blue R-250 staining (Cat. ZD305A, ZOMANBIO, China).

### SDS-PAGE and western blot analysis

Standard procedures for sodium dodecyl sulfate polyacrylamide gel electrophoresis (SDS-PAGE) were employed. Protein concentrations were quantified using the Thermo Scientific Pierce BCA Protein Assay Kit (Cat. 23 227, Thermo Fisher, USA), a high-precision, detergent-compatible assay for determining total protein concentration. Proteins were diluted in 5 × SDS-PAGE sample loading buffer and boiled at 95°C for 10 min. Proteins were separated by 15% polyacrylamide gels (Cat. E305, Vazyme Biotech, China) and electroblotted onto PVDF membranes with Western Rapid Transfer Buffer (Cat. P0572, Beyotime, China). Membranes were blocked in QuickBlock™ Blocking Buffer (Cat. P0252, Beyotime, China) for 15 min at room temperature followed by overnight incubation at 4°C with primary antibody diluted (1:3000) in primary antibody dilution buffer (Cat. P0256, Beyotime, China) and 1 h incubation with HRP-conjugated secondary antibody diluted at 1:5000 in secondary antibody dilution buffer (Cat. P0258, Beyotime, China). After incubation with a secondary antibody, the membranes were incubated with the SuperFemto ECL Chemiluminescence Kit (Cat. E423, Vazyme Biotech, China). Images were acquired using the Bio-RaDTM ChemiDoc™ Imaging System. The following antibodies were utilized: Outer membrane protein A (OmpA) Antibody (dilution 1:3000, Catalog No. abx345696, sourced from Abbexa, UK), DYKDDDDK Tag (D6W5B) Rabbit mAb (HRP Conjugate) (dilution 1:3000, Catalog No. 86 861, Cell Signaling Technology), His-Tag (D3I1O) XP® Rabbit mAb (HRP Conjugate) (dilution 1:3000, Catalog No. 12 688, Cell Signaling Technology, USA), Anti-rabbit IgG, HRP-linked Antibody(1:3000, Cat. 7074, Cell Signaling Technology, USA).

### Electrophoretic mobility shift assay (EMSA)

EMSA was performed as previously described [14]. The upstream region (from -464 to 0 bp) of the *virK* gene and a 16S rDNA fragment (251 bp, negative control) were amplified using specific primers (Supplementary Table S1). Binding reactions were performed by incubating the DNA fragments (0.3 pM) with 6 × His-tagged PhoP protein in a solution containing 10 mM Tris–HCl (pH 8.0), 25 mM MgCl_2_, 50 mM NaCl, 1 mM DTT, 1 mM EDTA, 0.01% Nonidet P40, and 10% glycerol, and incubated at room temperature for 20 min. The reaction mixtures were then loaded on a 8% non-denaturing PAGE gel in native PAGE electrophoresis buffer at 100 V. Bands were detected using Bio-Rad ChemiDoc^TM^ XRS + System.

### DNase I footprinting assay

The DNase I footprinting assays were performed to determine PhoP-binding sites as previously described with some modifications [15]. The target DNA fragment within the *virK* promoter region was PCR amplified using the primers listed in Supplementary Table S1, and the amplicon was used as the template for further preparation of fluorescent 5-carboxyfluorescein (FAM)-labeled probes. Probes were incubated with 0, 2, 5, and 10 µg of Flag-PhoP for 30 min at 25°C. Then 5 μL of a solution containing 10 U of DNase I (Cat. EN0523, Thermo Fisher, USA) and 1 mol of freshly prepared CaCl_2_ was added, followed by further incubation for 55 sec at 37°C. 10 μL of 0.5 M EDTA was added immediately after incubation, mixed well (within 65 s), and inactivated at 65°C for 10 min. Recovery of reaction products was extracted with phenol/chloroform and precipitated with ethanol. The pellets were dissolved in 30 µL MiniQ water, and then sequenced with 3130XL DNA analyzer by Sangon Biotech (Shanghai) Co., Ltd.

### Separation of inner and outer membranes by EDTA-free sucrose gradient centrifugation

Bacterial subcellular separation was carried out according to the procedures described in literatures [16, 17] with some modifications. The bacterial cultures were centrifuged at 5000 × *g* for 10 min at 4°C to harvest the cells, and the cell pellets were resuspended in 40 mL of lysis buffer (50 mM Tris–HCl, pH 7.8, 300 mM NaCl, 10% glycerol (v/v)) supplemented with UltraNuclease (25U/mL, Cat. 20156ES50, YEASEN). The cells were broken by French Press (GLEN MILLS, America) at 15 000 psi with two passes, and the suspensions were centrifuged at 5000 × *g* for 10 min at 4°C to remove cell debris. Total membranes were then isolated by centrifugation using an MLA-55 rotor in Optima^TM^ MAX-XP Ultracentrifuge (Beckman) at 185 000 × *g* for 2 h and resuspended in 20% (w/v) sucrose (20 mM Tris–HCl, pH 7.8). The inner and outer membranes were subsequently separated by the use of a defined 73–53–20% sucrose gradient through ultracentrifugation with no braking at 250 000 ×* g* for 18 h at 4°C using an MLS-50 rotor (Beckman). The upper IM and OM layers were collected using the pipettes and transferred into ultracentrifuge bottles. The dilution buffer (50 mM Tris–HCl, pH 7.8, 300 mM NaCl) was then added into the ultracentrifuge bottles to a final sucrose concentration of <10%. The IM and OM suspensions were ultracentrifuged in an MLA-55 rotor at 185 000 × *g* for 2 h at 4°C. The purity of the separated OM and IM was checked, and the subcellular localization of VirK protein was detected by western blot using anti-OM (OmpA) and anti-HIS proteins.

### Outer membrane permeability and inner membrane integrity assay

The fluorescent dyes N-phenyl-1-naphthylamine (NPN) and propidium iodide (PI) were used to evaluate the permeability of the bacterial outer membrane and the inner membrane integrity, respectively. The assay was performed as previously described [18, 19]. Briefly, overnight cultures of indicated strains were transferred to fresh LB and grown to an OD_600_ of 1.0. Bacterial cells were washed twice with 5 mM HEPES containing 5 mM glucose (pH 7.0) and resuspended in the HEPES buffer to an OD_600_ of 0.5. Bacterial suspensions were incubated with final doses of NPN (10 μM) and PI (10 μM and 20 μM) for different times. Using an EnSpire™ 2300 Multilabel Plate Reader (PerkinEImer) with excitation/emission wavelengths of 350 nm/420 nm (NPN) and 535 nm/615 nm (PI), the permeability of the outer/cell membrane was then measured.

### Growth curve

The fitness of deletion mutants was confirmed by analyzing growth curves to assess the growth rate and viability, complemented by transmission electron microscopy to examine cell shape changes. Cells of overnight cultures of bacteria grown in 3 mL CAMHB liquid medium at 37°C were harvested at 3000 × *g* for 5 min and washed twice with sterilized double-distilled H_2_O. The resuspended cultures were diluted at a 1:100 ratio into CAMHB liquid medium and grown at 37°C with shaking at 220 rpm. Growth was monitored by measuring OD_600_ every 1 h for 24 h by Bioscreen C°PRO.

### Lipid A characterization with ESI-TOF-MS

Lipid A extraction was performed as described before [10, 20, 21] with some modifications. Cell pellets were suspended in PBS, and chloroform and methanol were then added to the tube for a single-phase Bligh-Dyer mixture (chloroform/methanol/water, 1:2:0.8, v/v/v). After incubating at room temperature for 20 min, pellets were collected and resuspended in hydrolysis buffer (50 mmol/L sodium acetate, pH 4.5; 1% SDS), assisted by sonication at a constant duty cycle for 5 s at 25% output (5 s/burst, ∼5 s between bursts, 5 min in total), then keep in a water bath at 95°C for 1 h. The SDS solution was converted into a two-phase Bligh-Dyer mixture by adding chloroform and methanol to form a chloroform/methanol/water (2:2:1.8, v/v/v) mixture. The lower phases were collected and washed twice with the upper phase of a preequilibrated two-phase Bligh-Dyer mixture (2:2:1.8, v/v/v), followed by drying under nitrogen. The dried lipids were re-dissolved in chloroform/MeOH (1:1, v/v) and characterized by MALDI-TOF/TOF MS (AXIMA PerformanceTM, Shimadzu, Japan) in negative ion mode.

### Antibacterial activity assay

Minimum inhibitory concentrations (MICs) were determined using the broth microdilution (BMD) method according to EUCAST guidelines [22]. Briefly, fresh bacterial cultures were adjusted to 0.5 McFarland, diluted 1:100 in CAMHB, and inoculated (final inoculate dose of about 5 × 10^5^ CFU/mL) into a 96-well plate containing serial concentrations of polymyxin B, polymyxin S_2_, colistin, or LL-37 [23] diluted in CAMHB and incubated for 20–24 h at 37°C. The MIC was determined as the lowest concentration that inhibits bacterial growth. In vitro polymyxin resistance is defined as an MIC to polymyxin exceeding 2 μg/mL, as per the EUCAST breakpoint criteria.

### Statistical analysis

Statistical analysis was performed by GraphPad Prism. Statistical significance was analyzed using ordinary one-way ANOVA followed by Tukey’s multiple comparisons test. *P *< 0.05 was considered statistically significant. All experiments were repeated at least three times.

### Ethical approval statement

Animals had free access to food and water during the study. All the animal studies complied with the animal husbandry guidelines, and all animal experiments were performed according to national standards for laboratory animals in China (GB/T 35892–2018), with approval from the Laboratory Animal Welfare & Ethics Committee in the Institute of Medicinal Biotechnology, Chinese Academy of Medical Sciences & Peking Union Medical College (Approval No: IMB-20220915D902, IMB20260105D901).

## Results

### Correlation and function of VirK in VirK/YbjX family proteins

**Figure 1.**
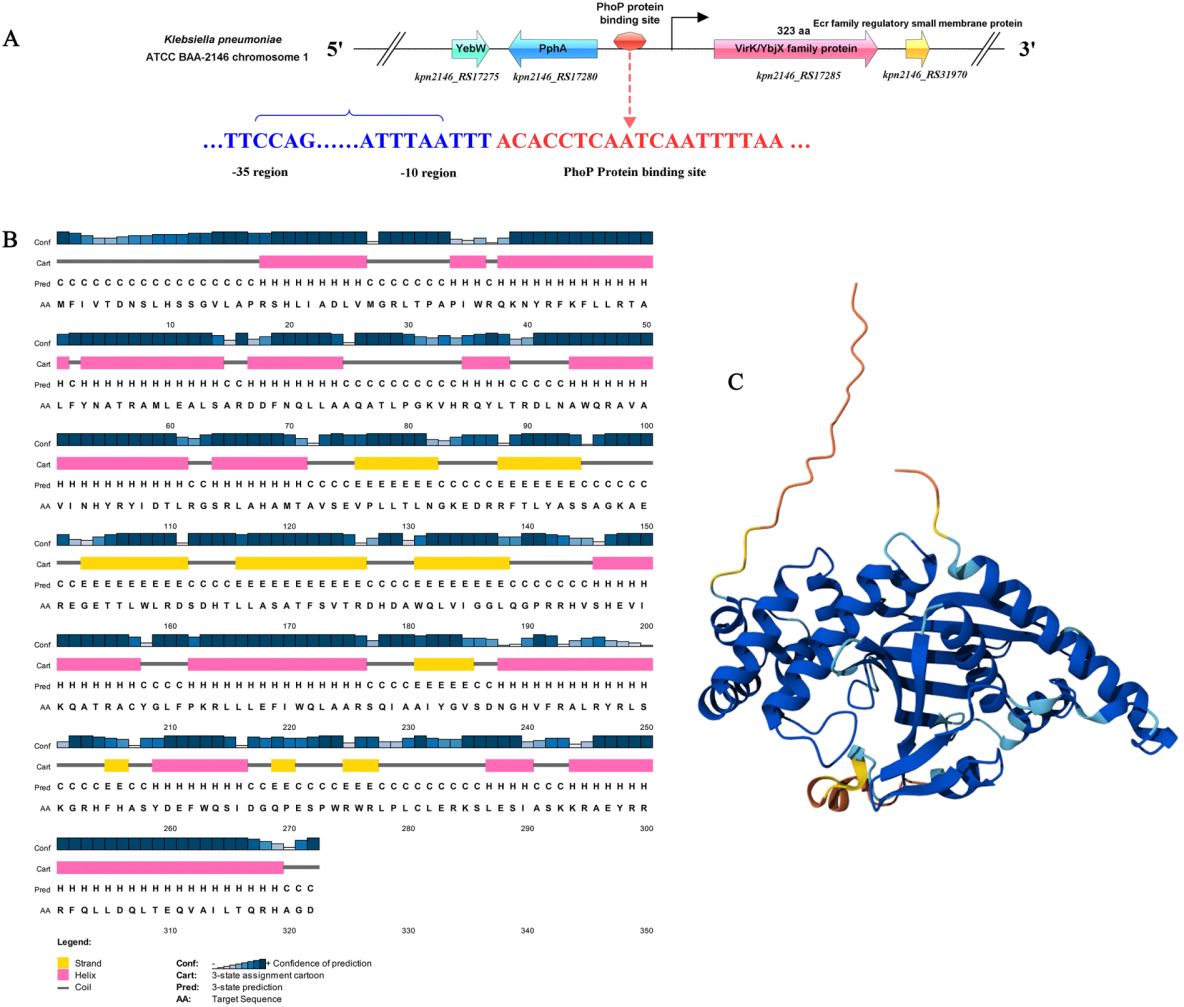
Identification, sequence characterization, and structural analysis of VirK in *K. pneumoniae*. (**A**) Schematic diagram of the sequence of *virK* in the upstream region. The predicted promoter is displayed in blue font, while the PhoP protein binding site is displayed in red font. (**B**) The secondary structure of VirK was predicted using PSIPRED, a widely recognized method for protein secondary structure prediction that uses the physical and chemical properties of the protein sequence to classify and determine the presence of α-helices, β-sheets, and random coils. Among them, the α-helix and the β-fold are represented by pink and yellow cylinders, respectively. (**C**) The predicted protein structure of VirK using AlphaFold (AFDB accession: AF-A6TB10-F1).

The *virK* gene in Mut-S is 969 bp in length and encodes a protein of 322 amino acids. The predicted -10 and -35 regions of the *virK* promoter are close to the transcription start site (Fig. 1A). The protein structure of VirK is predicted to be a helix-strand-strand-strand-helix fold according to PSIPRED [24] and AlphaFold [25], including fourteen α-helixes (α1–14) flanking nine β-sheets (β1–9) (Fig. 1B,C). Further comparison of the VirK homologs and other VirK/YbjX family members in the GenBank database revealed that VirK in *K. pneumoniae* exhibited 19∼23% similarity in terms of amino acids compared with other members of the VirK/YbjX family (Supplementary Fig. S1). All VirK homologs in the VirK/YbjX family possess the same conserved α-helix amino acid sequence (Supplementary Fig. S2).

**Figure 2.**
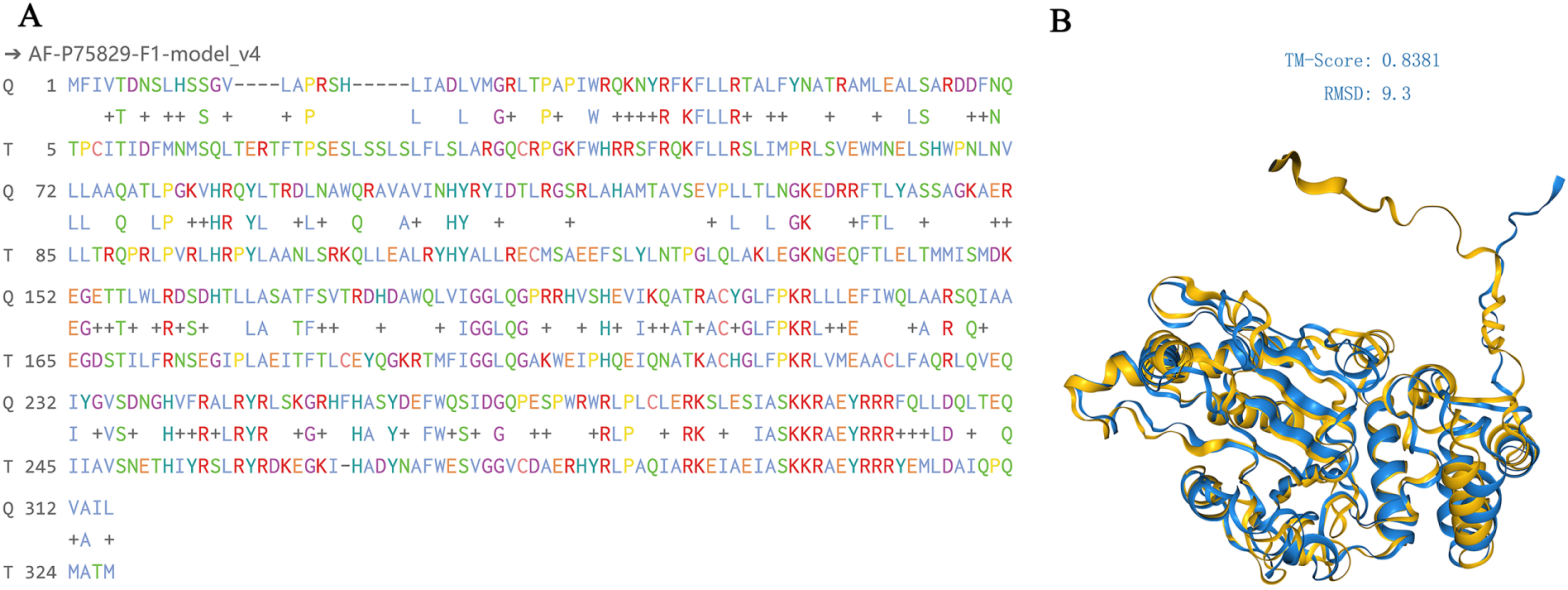
Sequence and structure comparison of VirK (from *K. pneumoniae* ATCC BAA2146) and YbjX (from *E. coli K-12*) proteins. (**A**) Alignment of the amino acid sequence of VirK (**Q**) with that of YbjX (**T**); (**B**) Comparison of the protein structures of VirK and YbjX. Amino acid sequences and structures were compared using the foldseek server [30]. The protein structure of YbjX was obtained from the AFDB database (AFDB accession: AF-P75829-F1).

YbjX, a member of the family of proteins of unknown function (DUF535), has been extensively studied in *E. coli* for its role in pathogenicity [26–29]. Comparison of VirK in *K. pneumoniae* with YbjX in *E. coli* revealed 33.92% amino acid similarity (Fig. 2A). Structural alignment revealed that the two proteins shared 322 residues, with a root mean square deviation (RMSD) of 9.3 Å for the common residues and a TM score of 0.8381. These metrics suggest a significant structural similarity between the two proteins, indicating that they adopt a similar overall fold (Fig. 2B).

### PhoP directly regulates the expression of *virK* in *K. pneumoniae*

**Figure 3.**
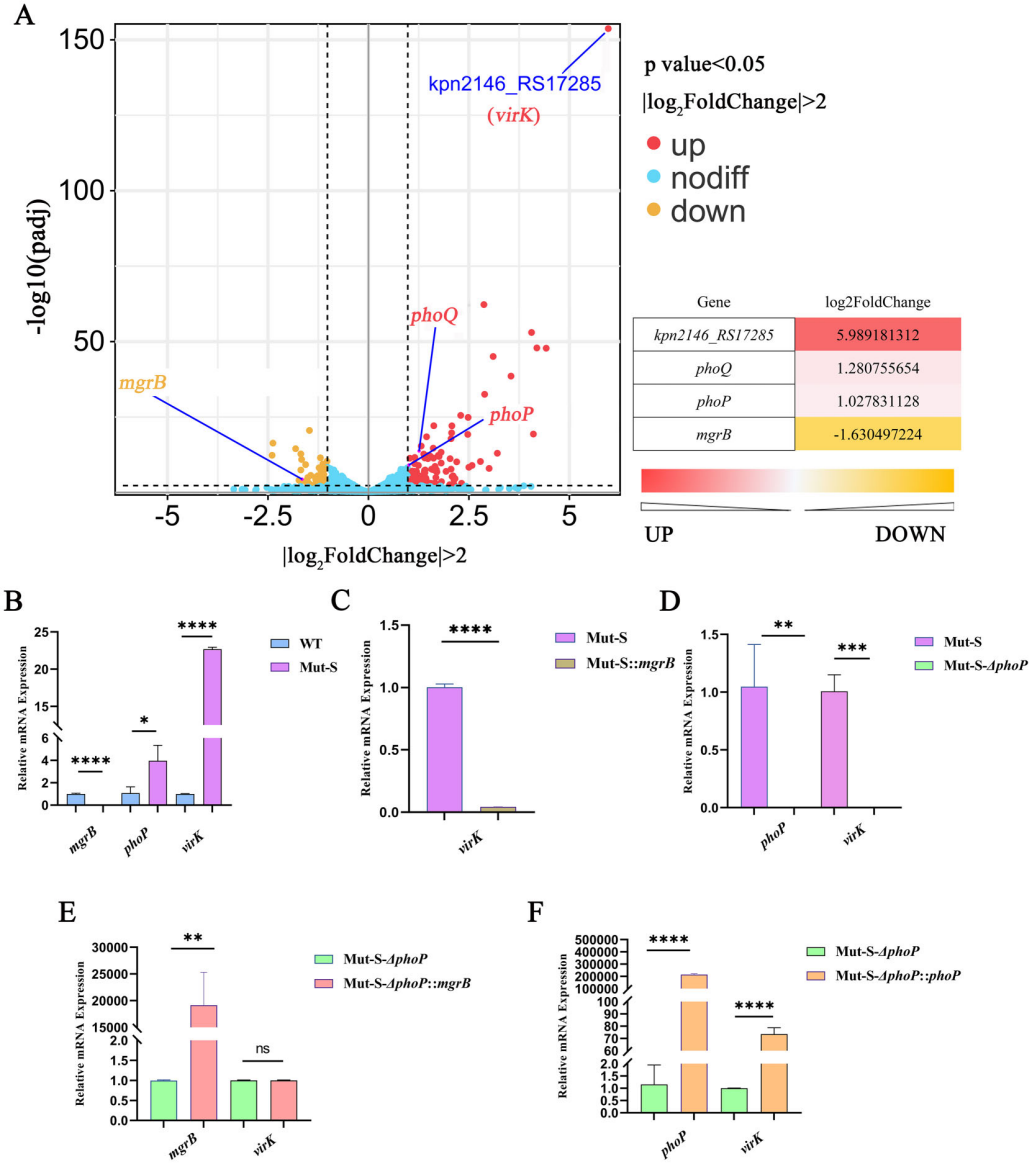
Transcriptomic analysis of Mut-S in comparison to Kpn2146 strains. (**A**) Volcano map displaying the upregulated (red dots) and downregulated (yellow dots) genes between the Mut-S group and the control group. (**B**) Changes in the relative expression of the *mgrB, phoP*, and *virK* genes in the Mut-S strain compared with those in the WT strain. (**C**)Changes in the *virK* gene expression in the Mut-S strain after supplementation with the *mgrB* gene. (**D**) Changes in *phoP* and *virK* gene expressions after *phoP* gene knockdown in the Mut-S strain. (**E**) Changes in the *mgrB* and *virK* gene expressions after the *mgrB* gene was complemented in the *phoP* knockout strain in Mut-S. (**F**) Changes in the *phoP* and *virK* gene expressions after the *phoP* gene was supplemented in the *phoP* knockout strains in Mut-S. *P < 0.05, **P < 0.01, ***P < 0.001, and ****P < 0.0001.

**Figure 4.**
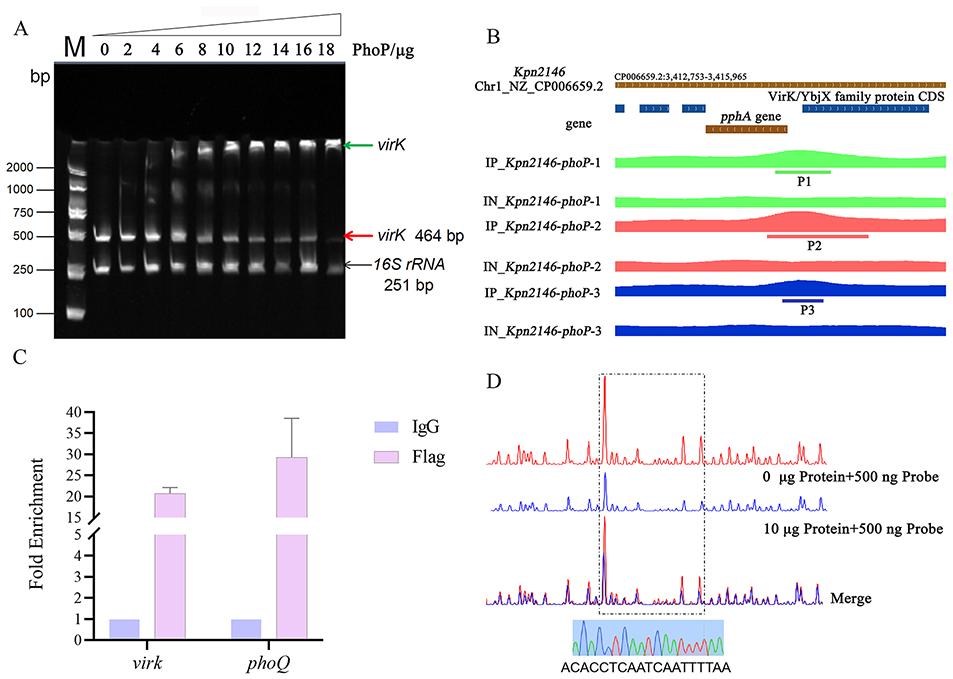
PhoP directly regulates the expression of *virK* in *K. pneumoniae*. (**A**) The ability of PhoP to bind to the *virK* promoter was determined by EMSA. His-tagged PhoP was incubated with *virK* and 16S rDNA (negative control) in a concentration gradient. The experiments were repeated three times. An upward arrow marks bound DNA fragments, a middle arrow indicates free DNA fragments, and the bottom arrow represents the 16S rDNA fragment.(**B**) Mapping of PhoP binding sites in *K. pneumoniae* ATCC BAA 2146 by ChIP-seq. IGV genome browser view showing PhoP binding regions around the *virK* gene across the *K. pneumoniae* genome. IP: Experimental group, namely the IP samples; IN: Control group, namely the IN sample; numbers represent three independent replicate trials. (**C**) ChIP and real-time PCR assays were used to investigate the binding of PhoP to the putative binding site in the promoter region of the *virK* gene. The experiments were independently repeated three times. The PhoQ gene was used as a positive control. The ChIP DNA was enriched using an IgG antibody and a Flag antibody and quantified by qPCR. (**D**) DNase I footprinting analysis of PhoP binding to the *virK* promoter. The intergenic fragment was labeled with 6-carboxyfluorescein (FAM) dye and incubated with 10 μg of PhoP (upper curve) or without PhoP (lower curve). The region protected by PhoP from DNase I cleavage is indicated with a black dotted box (ACACCTCAATCAATTTTAA).

Analysis of genes upregulated in Mut-S by RNA-seq revealed a significant increase in the expression of the PhoQ/PhoP TCS compared with Kpn2146. In addition to the increased expression of downstream PhoQ/PhoP TCS components and genes associated with polymyxin resistance, the expression of the *virK* gene with unknown function was abnormally elevated (log_2_FoldChange = 5.9891, *p *= 1.71E-154) (Fig. 3A). The expression of genes in different strains was further evaluated by RT‒qPCR. Compared with those in the wild-type strain, the expressions of *phoP* and *virK* in Mut-S were approximately 4-fold and 22-fold higher, respectively, while the expression of *mgrB* was downregulated [10] (Fig. 3B). Compared with Mut-S, the complementation of *mgrB* in Mut-S decreased the expression of *virK* (Fig. 3C). The knockout of *phoP* (Supplementary Fig. S3) in Mut-S resulted in decreased *virK* expression (Fig. 3D), and supplementation with *mgrB* did not change the expression of *virK* (Fig. 3E), whereas *phoP* supplementation resulted in increased*virK* expression and increased *phoP* expression (Fig. 3F). In addition to the conclusion that MgrB negatively regulates *phoP* gene expression, our experimental results suggest that PhoP directly regulates *virK* gene expression in *K. pneumoniae*. To further explore the regulatory mechanism of PhoP, the purified His-tagged PhoP protein was incubated with a 464 bp promoter fragment of the *virK* gene and subjected to electrophoretic mobility shift assay (EMSA), with a 251 bp sequence from the middle of the 16S rRNA serving as the negative control. EMSA revealed that PhoP binds efficiently to the *virK* promoter region *in vitro* in a concentration-dependent manner (0–18 μg). In contrast, the negative control 16S rRNA sequence showed no detectable binding even at the highest concentration tested (Fig. 4A).

To confirm that VirK is a key downstream factor regulated by the PhoP/PhoQ TCS and elucidate the underlying regulatory mechanism, we employed a chromatin immunoprecipitation sequencing (ChIP-seq) assay to identify the target genes directly regulated by the PhoP protein. To facilitate ChIP-seq of *K. pneumoniae* PhoP, we introduced N-terminal FLAG tags at the native *phoP* locus. Sequence reads were obtained from ChIP-seq analysis using an anti-Flag antibody and mapped to the *K. pneumoniae* ATCC BAA-2146 genome (ASM36438v3). After information on the effective reads aligned to the genome was obtained, MACS analysis software was used to analyze the peak information within the genome. The threshold for selecting significant peaks was a q value < 0.05. The ChIP-seq data revealed that PhoP bound to multiple gene promoters. Among the potential target genes, *phoQ, arnB, arnC, lpxB*, and *lpxC* were found to be associated with Lipid A synthesis and resistance to polymyxins in *K. pneumoniae*. In addition, three peaks (P1, P2, and P3) with partially overlapping regions were detected in the promoter region of the *virK* gene across three independent experiments (Fig. 4B). The sequences of these peaks are shown in the Supplementary Materials. To determine the binding of PhoP to the *virK* locus, a ChIP‒qPCR assay was used to assess the enrichment of PhoP at the *virK* site. The results indicated that the binding sequence recognized by the PhoP protein encompasses the promoter region of the *virK* gene (Fig. 4C), consistent with the ChIP-seq data.

Dye-primer-based DNase I footprinting analysis was further conducted to precisely locate the PhoP binding site within the *virK* promoter region. As depicted in Fig. 4D, a DNase I-hypersensitive site was mapped to nucleotides −96 bp to −77 bp upstream of the ATG start codon of the *virK* gene (ACACCTCAATCAATTTTAA), which is indicative of a regulatory region protected by the PhoP protein. This site matches the consensus sequence identified from the ChIP-seq data, confirming the importance of the DNA-binding ability of PhoP.

### VirK is located on the outer membrane of *K. pneumoniae*

**Figure 5.**
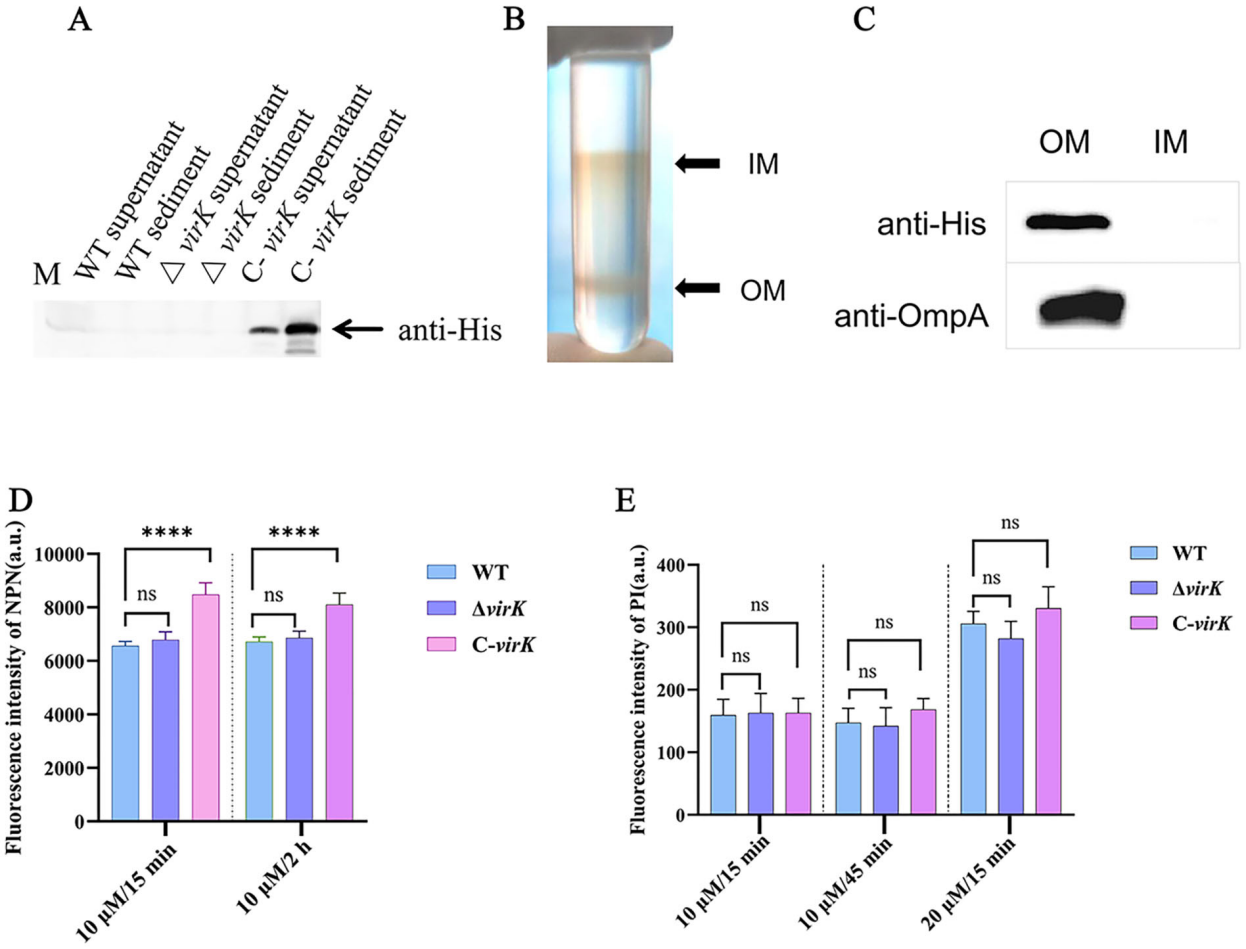
Subcellular localization of VirK protein in *K. pneumoniae*. (**A**) Detection of VirK protein using an anti-His-tag antibody in cellular fractions of different strains following bacterial cell disruption and centrifugation. (**B**) Images of discontinuous sucrose density gradients after isopycnic centrifugation of the C-*virK* supernatant. The upper band corresponds to the inner membrane (IM), while the lower band represents the outer membrane (OM). (**C**) Western blot detection of the VirK protein in the inner and outer membranes. Anti-OmpA was used as a loading control. NPN and PI uptake experiments to determine the membrane permeability of different strains. (**D**) 10 μM NPN treatment for 15 min and 2 h. (**E**) 10 μM PI treatment for 15 and 45 min, or 20 μM PI treatment for 45 min. The fluorescence intensity reflects the internal flow rate of NPN and PI, and the higher the value is, the stronger the membrane permeability and membrane damage. The data are presented as the mean ± SD (*n* = 9), with *P* values adjusted for multiple comparisons determined using one-way ANOVA. a.u., arbitrary units; ns, no statistically significant difference; ****, *P *< 0.0001.

To elucidate the function of *K. pneumoniae* VirK, its subcellular localization was subsequently examined. We generated a plasmid carrying a His-tagged VirK variant (VirK-His) and assessed its ability to rescue the *virK* deficiency after complementation. Western blot analysis revealed that the VirK-His protein was undetectable in both the supernatant and precipitate fractions following ultrasound-induced cell disruption in both the wild-type (Kpn2146) and the *virK* knockout (Kpn2146 Δ*virK)* strains. However, VirK-His was detected in both the supernatant and precipitate of the *virK* complementation strain (*C-virK*) (Fig. 5A). To probe the nature of the association of the VirK protein with the membrane, the bacterial membrane fraction of *C-virK* was subjected to further separation by sucrose gradient centrifugation without EDTA (Fig. 5B), and the presence of VirK-His in different fractions was detected by Western blotting, with OmpA (the known outer membrane-localized protein) as a positive control after SDS‒PAGE. Similar to OmpA, VirK-His was present in the outer membrane (OM), and no VirK-His or OmpA was detected in the inner membrane (IM) (Fig. 5C).

### VirK overexpression increases the outer membrane permeability of *K. pneumoniae*

When OM permeabilization was assessed via NPN assays, the fluorescence detection results showed that the deletion and complementation of the *virK* gene did not have a time-dependent effect on bacterial membrane permeability. After treatment with 10 μM NPN for 15 min and 2 h (Fig. 5D), the fluorescence intensity did not significantly differ between the Δ*virK* mutant strain (Kpn2146Δ*virK*) and the WT strain (Kpn2146) (*p *= 0.2514 at 15 min; *p *= 0.4950 at 2 h), whereas the fluorescence intensity of the *virK* complementation strain (C-*virK*) increased significantly (*p *< 0.0001 at both 15 min and 2 h). These results indicate that the loss of VirK function did not significantly alter membrane permeability, but the complementation strain may have triggered compensatory membrane structural changes because of the overexpression of *virK*. The deletion and complementation of the *virK* gene had no significant effect on PI intake (Fig. 5E). After treatment with 10 μM PI for 15 min, no statistically significant difference in fluorescence intensity was detected among the WT, Δ*virK* mutant, and C-*virK* strains (*p *= 0.9408 and 0.9442, respectively). When the processing time was extended to 45 min or the PI concentration was increased to 20 μM, still no significant difference was detected between the three groups (*p *= 0.1435 and 0.1245). To determine whether VirK alters membrane permeability independently of Lipid A modification, we performed NPN and PI uptake assays in the serial strains from Mut-S, which harbors pre-existing Lipid A modification. In the NPN assay, deletion of *virK* resulted in a modest reduction in outer membrane permeability compared with Mut-S at 10 μM for 15 min (*p *< 0.01). After prolonged incubation (10 μM, 2 h), the *virK*-complemented strain exhibited significantly higher NPN fluorescence than the deletion mutant (*p *< 0.0001), while no significant difference was observed relative to Mut-S (Supplementary Fig. S5A). These data indicate that VirK exerts a subtle effect on outer membrane permeability, without overriding the dominant contribution of Lipid A modification. In contrast, PI uptake assays revealed no consistent differences in inner membrane permeability among Mut-S, Mut-S-Δ*virK*, and Mut-S::*virK* across multiple conditions. A transient increase in PI fluorescence was observed in Mut-S::*virK* at 10 μM for 15 min (*p *< 0.01), but this effect was not maintained at longer incubation times or higher concentrations (Supplementary Fig. S5B). Collectively, these results demonstrate that VirK does not significantly affect inner membrane integrity and only modestly modulates outer membrane permeability in a Lipid A–modified background.

### VirK contributes to the virulence of *K. pneumoniae*

To investigate the impact of *virK* genes on virulence in *K. pneumoniae*, we assessed the pathogenicity of various strains using a mouse systemic infection model. After infection with solutions of different concentrations of bacterial suspensions (WT, Kpn2146Δ*virK*, and C-*virK*) (Supplementary Fig. S3), the deaths of the mice were recorded daily for seven days, and the LD_50_ and LD_99_ values were calculated using the Probit method. For the WT strain, the LD_50_ and LD_99_ values were 4.47 × 10^5^ and 3.72 × 10^6^ CFU/mouse, respectively, while the corresponding values for the Kpn2146Δ*virK* strain were 8.51 × 10^5^ and 1.07 × 10^8^ CFU/mouse, respectively, indicating a modest but statistically significant attenuation of virulence following *virK* knockout. After complementing with the pTOPO-Apr-*virK* plasmid (the *C-virK* strain), the virulence of the strain was partially recovered, with the LD_50_ and LD_99_ being 1.00 × 10^6^ and 1.26 × 10^7^ CFU/mouse, respectively (Table 1). The partial restoration of virulence in the complemented strain suggests that VirK may not be solely responsible for full pathogenicity. To assess whether increased virulence and polymyxin resistance could coexist in the Mut-S background, we further evaluated the *in vivo* virulence of the Mut-S strain together with its *virK* deletion and complemented derivatives using a mouse infection model. As shown in Table 1, the Mut-S strain exhibited a relatively low LD₅₀ (1.95 × 10^6^ CFU/mouse), indicating high virulence. Deletion of *virK* in the Mut-S background resulted in an increased LD₅₀ (6.92 × 10^6^ CFU/mouse), suggesting attenuated virulence. Complementation of *virK* partially restored virulence, yielding an LD₅₀ of 1.00 × 10^6^ CFU/mouse. Although the observed differences in LD₅₀s were modest and the confidence intervals partially overlapped, these results indicate that *virK* contributes to virulence even in a polymyxin-resistant genetic background.

**Table 1.** Virulence comparison of the strains in the mouse systemic infection model

Strain	LD_50_ (CFU/ mouse)	95% confidence intervals	LD_99_ (CFU/ mouse)	95% confidence intervals
Kpn2146	4.47 × 10^5^	2.63 × 10^5^∼7.94 × 10^6^	3.72 × 10^6^	1.62 × 10^6^∼4.37 × 10^7^
Kpn2146Δ*virK*	8.51 × 10^5^	2.51 × 10^5^∼2.88 × 10^6^	1.07 × 10^8^	1.29 × 10^7^∼1.15 × 10^13^
*C-virK*	1.00 × 10^6^	5.37 × 10^5^∼1.82 × 10^6^	1.26 × 10^7^	4.90 × 10^6^∼1.91 × 10^8^
Mut-S	1.91 × 10^6^	1.10 × 10^6^∼3.47 × 10^6^	1.74 × 10^7^	8.71 × 10^6^∼5.89 × 10^7^
Mut-SΔ*virK*	6.92 × 10^6^	4.68 × 10^5^∼1.05 × 10^7^	7.94 × 10^7^	3.63 × 10^7^∼2.88 × 10^8^
Mut-S:: *virK*	1.55 × 10^6^	1.26 × 10^6^∼2.95 × 10^6^	1.38 × 10^7^	6.76 × 10^6^∼4.68 × 10^8^

**Figure 6.**
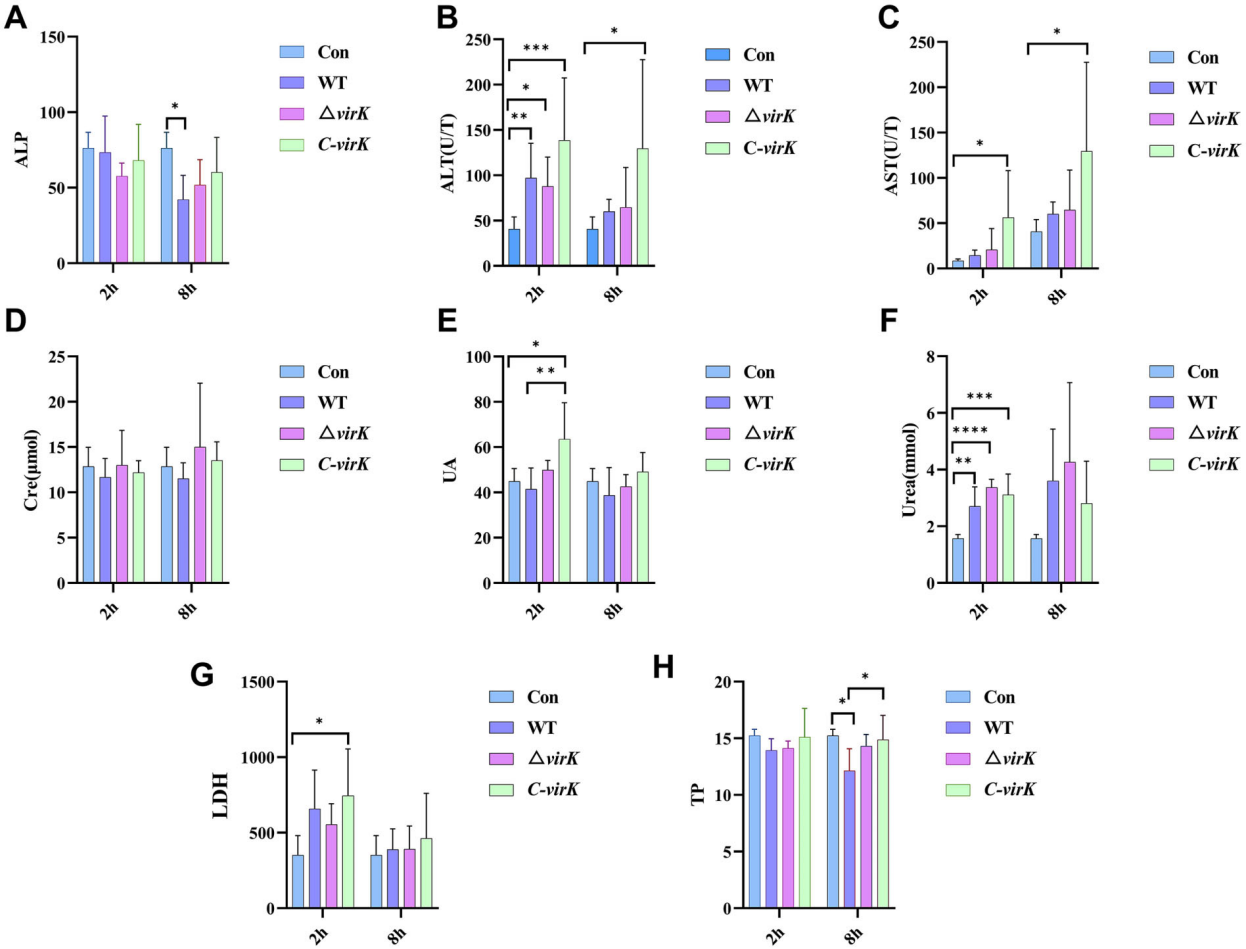
Serum biochemical indices of mice after infection; (**A**) Serum ALP levels at 2 and 8 h post-infection. (**B**) Serum ALT levels at 2 and 8 h post-infection. (**C**) Serum AST levels at 2 and 8 h post-infection. (**D**) Serum creatinine (Cre) levels at 2 and 8 h post-infection. (**E**) Serum uric acid (UA) levels at 2 and 8 h post-infection. (**F**) Serum urea levels at 2 and 8 h post-infection. (**G**) Serum LDH levels at 2 and 8 h post-infection. (**H**) Serum total protein (TP) levels at 2 and 8 h post-infection. The data are expressed as the means ± SDs (*n* = 6). Statistical significance was analyzed using ordinary one-way ANOVA followed by Tukey’s multiple comparisons test. *P < 0.05, **P < 0.01, ***P < 0.001.

Furthermore, Serum biochemical markers of the liver, including alanine aminotransferase (ALT), alkaline phosphatase (ALP), aspartate aminotransferase (AST), lactate dehydrogenase (LDH), total protein (TP) levels, creatinine (Cre), uric acid (UA), and urea levels, were measured in the different groups at 2 and 8 h postinfection. ALP levels did not significantly differ between the treatment groups (WT, Kpn2146Δ*virK*, and C-*virK*) and the control group at 2 h postinfection, whereas at 8 h, although all the treatment groups presented lower values than the control group did, only the WT group exhibited a statistically significant difference compared with the control group (Fig. 6A). At 2 h postinfection, ALT levels in the treatment groups were significantly higher than those in the control group (most pronounced in the C-*virK* group, *p *< 0.01), although no significant differences were observed among the treatment groups; at 8 h, ALT levels remained elevated in the treatment groups, with only the C-*virK* group maintaining a significant difference compared with the control group (*p *< 0.05; Fig. 6B). Although AST levels tended to increase overall, compared with the control group, only the C-*virK* group showed significant differences at 2 and 8 h after infection (*p *< 0.01, *p *< 0.05; Fig. 6C). There was no change in Cre levels within the treatment groups or between the treatment and the control groups (Fig. 6D). UA (Fig. 6E) and Urea (Fig. 6F) levels showed a similar increasing trend after 2 h of infection, and there was a significant difference between the C-*virK* group and the control group (*p *< 0.05, *p *< 0.0005). The LDH level tended to increase only at 2 h after infection, similar to the AST trend, and there was a statistically significant difference between the C-*virK* group and the control group (*p *< 0.05; Fig. 6G). Serum total protein (TP) levels at 2 and 8 h post-infection with Con, WT, Δ*virK*, and C-*virK* strains. No significant differences were observed at 2 h. At 8 h, TP levels were significantly lower in WT compared to Δ*virK* and C-*virK*, with complementation restoring TP levels (*P < 0.05; ordinary one-way ANOVA).

### *virK* gene does not affect the susceptibility of *K. pneumoniae* to polymyxins or LL-37

To investigate the relationship between the *virK* gene and polymyxin resistance, we tested the effects of *virK* gene knockout and complementation on polymyxin susceptibility. As indicated in Table 2, the *virK* gene does not influence the polymyxin MICs of strain Kpn2146, with MIC values ranging from 0.125 to 0.25 μg/mL for different polymyxins against Kpn2146, Kpn2146Δ*virK*, and *C-virK*. The Mut-S strain, which exhibits elevated *virK* expression, showed high-level resistance to polymyxins (polymyxin B and colistin), with MICs of 16–32 μg/mL, representing a 128–256-fold increase compared to the susceptible wild-type strain (Kpn2146). Moreover, knockout of the *virK* gene in Mut-S did not change its susceptibility to polymyxins. Similarly, the change in the *virK* gene did not affect the sensitivity of the strains to LL-37 in *K. pneumoniae*.

**Table 2.** Antimicrobial susceptibility of *K. pneumoniae* with different *virK* gene states to polymyxins

Strains	MICs (μg/mL)^a^			
	PXB	CST	PXS_2_	LL-37
Kpn2146	0.25	0.25	0.125	128
Kpn2146Δ*virK*	0.25	0.125	0.25	256
*C-virK*	0.125	0.25	0.25	128
Mut-S	32	16	32	—
Mut-SΔ*virK*	32	16	32	—

Abbreviations: PXB, polymyxin B; CST, colistin; PXS_2_, polymyxin S_2_; Mut-S: polymyxin S_2_-resistant Kpn2146; LL-37: a 37-residue amphipathic cathelicidin-derived antimicrobial peptide;

^a^MICs (minimum inhibitory concentrations) were determined by the broth microdilution method as recommended by CLSI[31].

### VirK expression does not modify the lipid A moiety of LPSs

**Figure 7.**
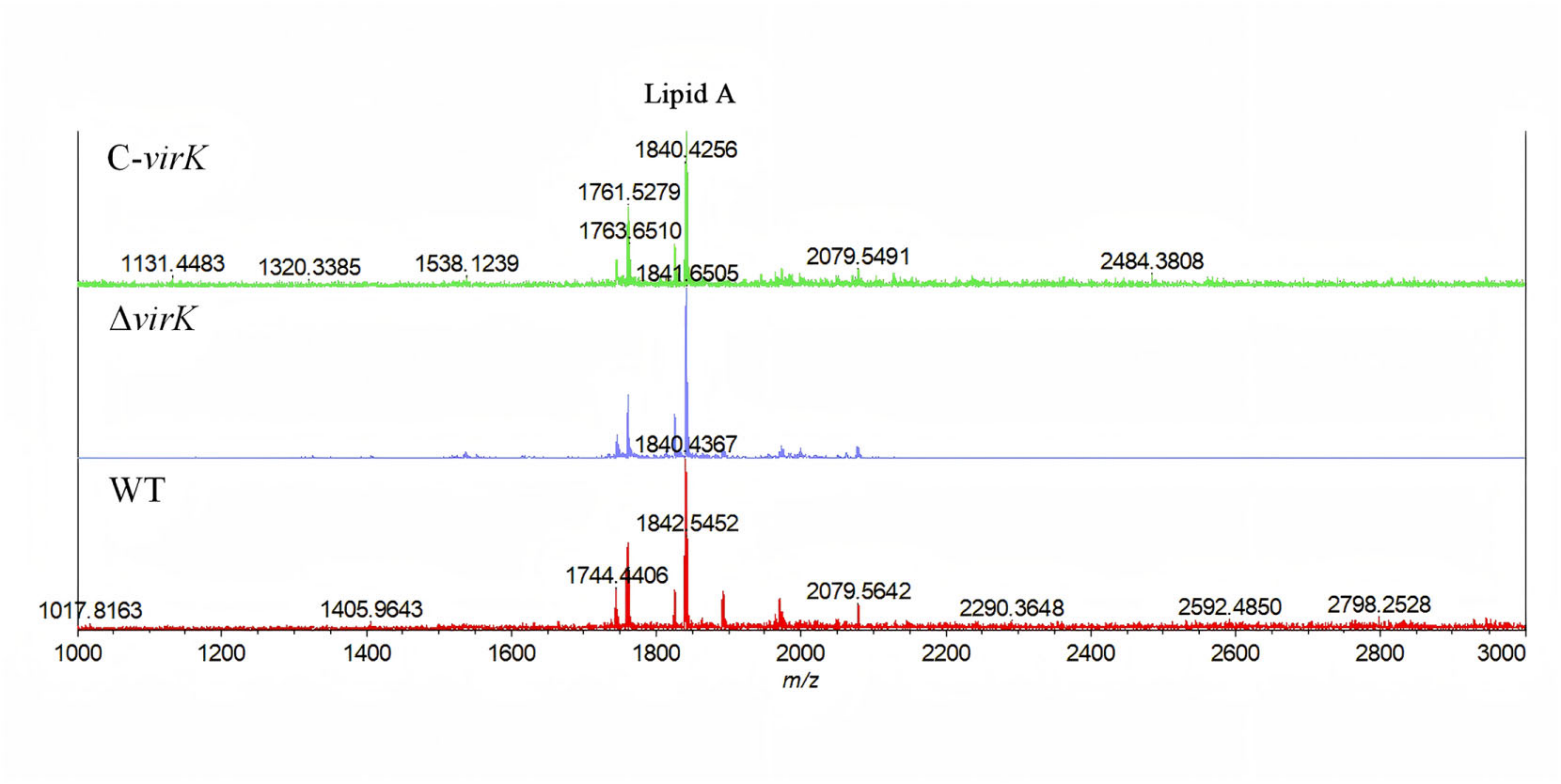
MALDI‒TOF–MS analysis of lipid A in *K. pneumoniae* strains with different expression states of *virK* genes. Negative ion MALDI‒TOF mass spectrum of the lipid A mixture. Spectra obtained from the average of >300 shots.

The above results confirmed that the PhoP protein regulates *virK* gene expression in *K. pneumoniae*. PhoP downstream regulatory proteins are involved in the modification of Lipid A. We analyzed the lipid A structures isolated from WT, Kpn2146Δ*virK*, and C-*virK* strains via mass spectrometry to investigate the possible chemical modification of *K. pneumoniae* LPS by VirK. The characteristics of the LPSs isolated from the WT, Kpn2146Δ*virK*, and C-*virK* strains were similar (Fig. 7), suggesting that VirK does not significantly affect the acylation profile of lipid A.

### High expression of the *virK* gene in clinically isolated polymyxin-resistant *K. pneumoniae*

**Figure 8.**
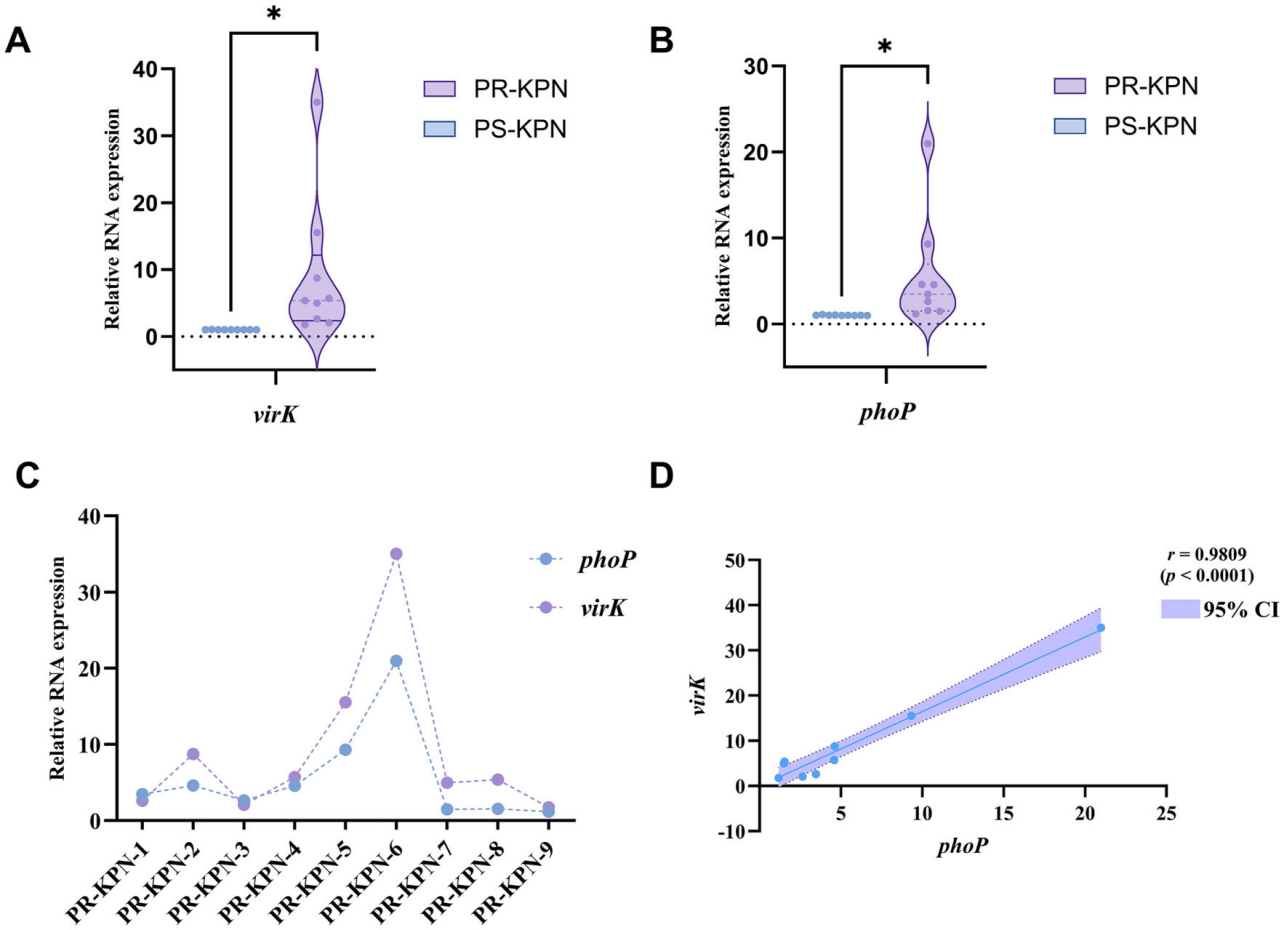
Analysis of *virK and phoP* gene expression in clinical *K. pneumoniae* isolates (PS-KPN and PR-KPN*)*. (**A**) and (**B**) Changes in the relative expressions of the *phoP* and *virK* genes in PR-KPN compared with those in PS-KPN. (**C**)and (**D**) Correlations between the expressions of the *virK* and *phoP* genes in the PR-KPN strains. The data are presented as the mean ± SD (*n* = 9), with *P* values adjusted for multiple comparisons determined using one-way ANOVA. a.u., arbitrary units; ns, no statistically significant difference; *P < 0.05.

We confirmed in the aforementioned results that the *virK* gene in polymyxin-resistant *K. pneumoniae* induced in the laboratory is regulated by the *phoP* gene. To verify whether the *virK* gene is also highly expressed in clinical strains of polymyxin-resistant *K. pneumoniae* (PR-KPN), we selected nine strains each of polymyxin-sensitive *K. pneumoniae* (PS-KPN) and PR-KPN (colistin MIC > 8) from the China CRE Network. Reverse transcription quantitative PCR (RT–qPCR) analysis showed that compared with those of the PS-KPN isolates, the expression levels of the *phoP* and *virK* genes of the PR-KPN strains significantly increased (Fig. 8A,B). Moreover, within the PR-KPN strains, a strong positive correlation was observed between the transcriptional profiles of *virK* and *phoP* (Fig. 8C), as evidenced by Pearson’s correlation analysis (*r* = 0.9809, *p *< 0.0001; Fig. 8D). The strong positive correlation between PhoP and VirK expression in clinical isolates is consistent with the regulatory relationship observed in laboratory strains, although expression correlation alone does not establish direct regulatory causality in clinical backgrounds.

## Discussion

Previous research has suggested that the *virK* gene may be regulated by phosphorylated PhoP protein in *Salmonella* [9, 32, 33]. Similarly, our preliminary transcriptome analysis of the Mut-S strain revealed that the *mgrB* gene was truncated, leading to increased expression of PhoQ/PhoP TCS downstream genes (*arnB, arnC, arnT*, etc.) and the *virK* gene [10], suggesting the possible regulatory function of the PhoQ/PhoP TCS on *virK* in *K. pneumoniae*. The RT‒qPCR results revealed that with functional *phoP*, the expression of *virK* increased simultaneously with *mgrB* truncation, indicating a correlation between the *virK* gene and the *mgrB* gene. However, following *phoP* gene knockout, complementation of the *mgrB* gene had no effect on the expression of the *virK* gene, whereas complementation of the *phoP* gene restored the upregulated expression of the *virK* gene in the *mgrB* mutant (Fig. 3). Therefore, we speculate that the *virK* gene in *K. pneumoniae* is also regulated by the PhoQ/PhoP TCS pathway, as are *arn/pmr* genes for polymyxin resistance. Through ChIP-seq and EMSA experiments, we confirmed that the PhoP protein was directly bound to the *virK* gene promoter region (-96 to -77 bp) and significantly increased its expression through positive regulation (Fig. 4). Hence, PhoP activation resulting from *mgrB* deficiency appears to be a significant contributor to the upregulation of *virK* in Mut-S, as indicated by the data in Fig. 3. This discovery extends our understanding of the PhoQ/PhoP regulatory network: in addition to classical target genes such as the *arn* and *pmr* operons, PhoP can also indirectly affect virulence by regulating the *virK* gene.

The elevated expression of *virK* observed in clinically isolated PS-KPN parallels the high expression of *phoP*, suggesting that the regulatory relationship defined in laboratory strains may also operate in clinical isolates, although causality cannot be inferred from expression correlation alone. Given the potential diversity in regulatory mutations among clinical isolates, including alterations in *phoPQ* or *mgrB*, these data can be interpreted as supportive rather than definitive evidence of a conserved regulatory mechanism. These findings indicate that the development of polymyxin resistance and enhanced virulence in clinical strains are not independent events; instead, increased virulence may naturally co-occur with the acquisition of antibiotic resistance.

The *virK* homologous gene is present in different Enterobacteriaceae [34]. Some studies have suggested that *Shigella flexneri* VirK and its homolog in *S. Typhimurium* may be involved in the remodeling of the bacterial envelope [7, 35]. VirK is peripherally associated with the cytoplasmic side of the plasma membrane in *Campylobacter jejuni* [6], and VirK is a periplasmic protein that is required for efficient secretion of plasmid-encoded toxins from enteroaggregative *E. coli* [34]. No studies have specifically investigated the localization of any VirK/YbjX family member in *K. pneumoniae* or how these proteins may function. Our results confirmed that VirK is located on the OM of *K. pneumoniae*, a structure characterized by its asymmetric composition of LPS and proteins. This positioning may be closely related to the function of VirK in virulence regulation, as the outer leaflet of the OM, where LPS predominantly resides, is crucial for the interaction of the bacterium with its environment. Outer membrane proteins commonly engage in interactions with the host pathogen, including adhesion, immune evasion, and the secretion of virulence factors [36–38]. Despite both VirK and YbjX belonging to the DUF535 family and exhibiting structural similarities [39], their functions differ significantly. YbjX enhances host defense resistance through myristic modification of lipid A, whereas VirK does not exhibit a chemical modification ability toward lipid A or LPS (Fig. 7). However, VirK overexpression increased the outer membrane permeability of *K. pneumoniae* (Fig. 5), suggesting the involvement of other unidentified mechanisms. VirK may increase virulence via mechanisms independent of LPS, such as through membrane protein localization or by interacting with other virulence determinants, as VirK in Shigella promotes intracellular transmission by regulating the membrane localization of the IcsA protein [8], which may delineate promising avenues for future research. In addition, the expression of VirK did not affect polymyxin susceptibility (Table 2), supporting the conclusion that PhoP affects polymyxin resistance and virulence involves parallel pathways rather than direct associations.

While elevated expression of the *phoP* gene in *K. pneumoniae* is frequently associated with LPS modification-mediated resistance [40], no consensus has been established regarding its impact on virulence [41–43]. Our data instead identify VirK as a specific effector of the virulence arm of the PhoP regulon. The effects of *virK* on bacterial pathogenicity were further evaluated in a mouse systemic infection model using *virK* mutants and complemented strains under *K. pneumoniae* ATCC BAA2146 (wild type) or Mut-S (*mgrB* truncated strain) backgrounds. Results from *K. pneumoniae* ATCC BAA2146 serial strains suggested that *virK* deficiency resulted in a modest reduction in bacterial virulence, while complementation partially restored this phenotype. These findings indicate that VirK functions as a contributing virulence factor rather than a dominant determinant of pathogenicity. Results from Mut-S serial strains showed that *virK* deletion resulted with a more profound effect on virulence, and complementation almost restored the pathogenicity of the strain, suggesting the accessory function of *virK* on virulence in polymyxin-resistant *K. pneumoniae*. The elevated expression pattern of *virK* observed in the transcriptome data suggested that VirK could increase virulence through the following mechanisms: (1) regulating outer membrane protein assembly and reducing the permeability of host antimicrobial peptides (such as LL-37); (2) interfering with the host’s early immune response signaling pathway; and (3) mediating the surface localization of virulence factors. Notably, VirK did not affect the susceptibility of the strain to LL-37 (Table 2) in our study, suggesting that its effect may focus mainly on immune escape rather than direct antimicrobial peptide resistance.

Previous studies have reported conflicting effects of *mgrB* inactivation on the virulence of *K. pneumoniae*, ranging from increased virulence to attenuation or no apparent change. Such discrepancies are likely attributable to strain-specific genetic backgrounds and differential downstream outputs of PhoP/PhoQ activation. While constitutive PhoP activation in *mgrB* mutants consistently promotes polymyxin resistance via lipid A modification, its impact on virulence appears more context dependent. Our findings indicate that PhoP activation also upregulates *virK*, a member of the VirK/YbjX family, which does not contribute to polymyxin resistance but modestly enhances virulence. We propose that variable engagement of PhoP-regulated virulence-associated factors such as *virK*, together with differences in strain background and experimental infection models, may underlie the previously reported inconsistencies in the virulence phenotypes of *mgrB* mutants.

The TCS, which is composed of a sensor histidine kinase and a response regulator, is an important bacterial regulatory system in response to external stimuli. In *K. pneumoniae*, the TCS mediates virulence traits, antibiotic resistance pathways, and stress survival mechanisms [44]. Virulence factor expression in *K. pneumoniae* has been reported to be regulated by several TCSs, including RcsAB, KvgSA, KvhSA, BarA/UvrY, EnvZ/OmpR, and CrrAB QseBC. These two-component systems can regulate the expression of virulence factors that are involved mainly in the regulation of pili, capsule polysaccharides (CPSs), lipopolysaccharides (LPSs), and iron carriers. PhoP/PhoQ is involved mainly in regulating the resistance of *K. pneumoniae* to polymyxin [45]. Previous reports on the relationship between the phoP/phoQ two-component system and virulence have often referred to the regulation of lipid A-modified gene expression, which indirectly affects virulence. However, there is currently no consensus on this viewpoint. Although it is generally believed that the biological costs associated with drug resistance may enable benign susceptible bacteria to overcome resistance when antibiotic pressure is reduced, some studies have shown that compared with their susceptible counterparts, polymyxin-resistant bacteria are associated with higher virulence and greater fitness [46]. Rather, the retention of fitness appeared to be influenced more by specific strain backgrounds, with some strains being capable of accommodating different resistance mutations with no significant loss of virulence [47]. However, decreased virulence and fitness were observed in another study [48]. The truncation of *mgrB* in the previously studied Mut-S strain led to high expression of *phoP* and the downstream *virK* gene, which eventually led to high resistance of the strain to polymyxin and a corresponding increase in virulence [10]. The modification of lipid A weakens or eliminates TLR4 signaling and alters downstream cytokine profiles, allowing bacteria to evade the immune system and establish infection, conferring the ability of gram-negative bacteria to survive within the host [42]. However, lipid A modification of colistin-resistant *K. pneumoniae* did not alter the innate immune response in a mouse model of pneumonia, and other mechanisms have been proposed to outweigh the effects of these lipid A mutations in LPS [41]. Our research may provide a new perspective, as high expression of the *virK* gene alone caused increased strain virulence and a reduced growth rate (Supplementary Fig. S4).

In conclusion, we systematically investigated the function and regulatory mechanism of the *virK* gene in *K. pneumoniae*, revealing its role as an accessory factor contributing to bacterial virulence in polymyxin-resistant *K. pneumoniae*. We elucidated the direct regulatory effect of the PhoP/PhoQ two-component system on *virK*. Furthermore, we revealed that VirK does not play a role in LPS modification, in stark contrast to the function of YbjX, highlighting the functional diversity of the DUF535 family of proteins. In addition, although some studies have suggested that polymyxin resistance mutations may be accompanied by virulence attenuation [5, 47], the high expression of *virK* mediated by PhoP in this study suggests that elevated VirK expression may help maintain virulence in resistant strains rather than acting as a primary virulence driver, and clinically resistant strains may achieve an “adaptive advantage” through such mechanisms. These findings suggest that inhibitors aimed at the PhoP/VirK pathway could play a pivotal role in prevention and control strategies against drug-resistant bacteria by potentially reducing both bacterial resistance and virulence.

## Supplementary Material

gkag290_Supplemental_Files

## Data Availability

The RNA-Seq and ChIP-Seq raw data have been deposited in the NCBI SRA(Sequence Read Archive) database under the accession numbers PRJNA1355483 and PRJNA1346843, respectively. In addition, the supporting experimental datasets have been deposited in Figshare, and the links are as follows: https://figshare.com/s/dc1cb87d695750e7508d; https://figshare.com/s/c3076807f10bc294660e; https://figshare.com/s/eeef33e97986683f1da1; https://figshare.com/s/3ec17e0df2dacc7eeae1; https://figshare.com/s/a7d1d7371fad8aeaa65d.

## References

[1] Mao M, He L, Yan Q. An updated overview on the bacterial PhoP/PhoQ two-component signal transduction system. Front Cell Infect Microbiol. 2025;15:1509037. 10.3389/fcimb.2025.1509037 39958932 PMC11825808

[2] Ezadi F, Ardebili A, Mirnejad R et al. Antimicrobial susceptibility testing for polymyxins: challenges, issues, and recommendations. J Clin Microbiol. 2019;57:e01390–18. 10.1128/JCM.01390-18 30541939 PMC6440778

[3] McConville TH, Annavajhala MK, Giddins MJ et al. CrrB positively regulates high-level polymyxin resistance and virulence in *Klebsiella pneumoniae*. Cell Rep. 2020;33:108313. 10.1016/j.celrep.2020.108313 33113377 PMC7656232

[4] Kidd TJ, Mills G, Sa-Pessoa J et al. A *Klebsiella pneumoniae* antibiotic resistance mechanism that subdues host defences and promotes virulence. EMBO Mol Med. 2017;9:430–47. 10.15252/emmm.201607336 28202493 PMC5376759

[5] Arena F, Henrici De Angelis L, Cannatelli A et al. Colistin resistance caused by inactivation of the MgrB regulator is not associated with decreased virulence of sequence type 258 KPC carbapenemase-producing *Klebsiella pneumoniae*. Antimicrob Agents Chemother. 2016;60:2509–12. 10.1128/AAC.02981-15 26824959 PMC4808163

[6] Novik V, Hofreuter D, Galan JE. Characterization of a *Campylobacter jejuni* VirK protein homolog as a novel virulence determinant. Infect Immun. 2009;77:5428–36. 10.1128/IAI.00528-09 19797067 PMC2786475

[7] Wing HJ, Goldman SR, Ally S et al. Modulation of an outer membrane protease contributes to the virulence defect of *Shigella flexneri* strains carrying a mutation in the *virK* locus. Infect Immun. 2005;73:1217–20. 10.1128/IAI.73.2.1217-1220.2005 15664968 PMC547015

[8] Nakata N, Sasakawa C, Okada N et al. Identification and characterization of *virK*, a virulence-associated large plasmid gene essential for intercellular spreading of *Shigella flexneri*. Mol Microbiol. 1992;6:2387–95. 10.1111/j.1365-2958.1992.tb01413.x 1406277

[9] Detweiler CS, Monack DM, Brodsky IE et al. *virK, somA* and *rcsC* are important for systemic *Salmonella enterica* serovar Typhimurium infection and cationic peptide resistance. Mol Microbiol. 2003;48:385–400. 10.1046/j.1365-2958.2003.03455.x 12675799

[10] Li HB, Sun L, Qiao H et al. Polymyxin resistance caused by large-scale genomic inversion due to IS*26* intramolecular translocation in *Klebsiella pneumoniae*. Acta Pharmaceutica Sinica B. 2023;13:3678–93. 10.1016/j.apsb.2023.06.003 37719365 PMC10501869

[11] Wang Y, Wang S, Chen W et al. CRISPR-Cas9 and CRISPR-assisted cytidine deaminase enable precise and efficient genome editing in *Klebsiella pneumoniae*. Appl Environ Microb. 2018;84:e01834–18. 10.1128/AEM.01834-18 PMC6238054 30217854

[12] Concordet JP, Haeussler M. CRISPOR: intuitive guide selection for CRISPR/Cas9 genome editing experiments and screens. Nucleic Acids Res. 2018;46:W242–5. 10.1093/nar/gky354 29762716 PMC6030908

[13] Li X, Yin W, Lin JD et al. Regulation of the physiology and virulence of *Ralstonia solanacearum* by the second messenger 2′,3′-cyclic guanosine monophosphate. Nat Commun. 2023;14:7654. 10.1038/s41467-023-43461-2 37996405 PMC10667535

[14] Hellman LM, Fried MG. Electrophoretic mobility shift assay (EMSA) for detecting protein–nucleic acid interactions. Nat Protoc. 2007;2:1849–61. 10.1038/nprot.2007.249 17703195 PMC2757439

[15] Peek J, Lilic M, Montiel D et al. Rifamycin congeners kanglemycins are active against rifampicin-resistant bacteria via a distinct mechanism. Nat Commun. 2018;9:4147. 10.1038/s41467-018-06587-2 30297823 PMC6175910

[16] Shu S, Mi W. Separating inner and outer membranes of *Escherichia coli* by EDTA-free sucrose gradient centrifugation. Bio-protocol. 2023;13:e4638. 10.21769/BioProtoc.4638 36968434 PMC10031520

[17] Shu S, Mi W. Regulatory mechanisms of lipopolysaccharide synthesis in *Escherichia coli*. Nat Commun. 2022;13:4576. 10.1038/s41467-022-32277-1 35931690 PMC9356133

[18] Cai J, Shi J, Chen C et al. Structural-activity relationship-inspired the discovery of saturated fatty acids as novel colistin enhancers. Adv Sci. 2023;10:e2302182. 10.1002/advs.202302182 PMC10582468 37552809

[19] Yang B, Liu C, Pan X et al. Identification of novel *phoP-phoQ* regulated genes that contribute to polymyxin B tolerance in *Pseudomonas aeruginosa*. Microorganisms. 2021;9:344. 10.3390/microorganisms9020344 33572426 PMC7916210

[20] Hankins JV, Madsen JA, Needham BD et al. The outer membrane of Gram-negative bacteria: lipid A isolation and characterization. Methods Mol Biol. 2013;966:239–58. 10.1007/978-1-62703-245-2_15 23299739 PMC4919815

[21] Caroff M, Novikov A. In: Holst O (ed.), Microbial Toxins: Methods and Protocols. Totowa, NJ: Humana Press, 2011, 135–46. 10.1007/978-1-61779-102-4_12

[22] The European Committee on Antimicrobial Susceptibility Testing . Breakpoint tables for interpretation of MICs and zone diameters. Version 16.0. https://www.eucast.org (25 January 2026, date last accessed).

[23] Friberg C, Haaber JK, Vestergaard M et al. Human antimicrobial peptide, LL-37, induces non-inheritable reduced susceptibility to vancomycin in *Staphylococcus aureus*. Sci Rep. 2020;10:13121. 10.1038/s41598-020-69962-4 32753585 PMC7403302

[24] McGuffin LJ, Bryson K, Jones DT. The PSIPRED protein structure prediction server. Bioinformatics. 2000;16:404–5. 10.1093/bioinformatics/16.4.404 10869041

[25] Jumper J, Evans R, Pritzel A et al. Highly accurate protein structure prediction with AlphaFold. Nature. 2021;596:583–9. 10.1038/s41586-021-03819-2 34265844 PMC8371605

[26] Song XJ, Li CX, Qi KZ et al. The role of the outer membrane protein gene in the pathogenicity of avian pathogenic. Avian Pathol. 2018;47:294–9. 10.1080/03079457.2018.1448053 29517278

[27] Song X, Qiu M, Jiang H et al. *ybjX* mutation regulated avian pathogenic Escherichia coli pathogenicity though stress-resistance pathway. Avian Pathol. 2019;49:144–52. 10.1080/03079457.2019.1687844 31670582

[28] Song XJ, Hou MM, Tu J et al. Outer membrane proteins YbjX and PagP co-regulate motility in via the bacterial chemotaxis pathway. Res Vet Sci. 2019;125:279–84. 10.1016/j.rvsc.2019.07.008 31326704

[29] Marciano DC, Wang C, Hsu T-K et al. Evolutionary action of mutations reveals antimicrobial resistance genes in *Escherichia coli*. Nat Commun. 2022;13:3189. 10.1038/s41467-022-30889-1 35680894 PMC9184624

[30] van Kempen M, Kim SS, Tumescheit C et al. Fast and accurate protein structure search with Foldseek. Nat Biotechnol. 2024;42:243–6. 10.1038/s41587-023-01773-0 37156916 PMC10869269

[31] CLSI. *Performance Standards for Antimicrobial Susceptibility Testing*. 35th ed. CLSI supplement M100. (ISBN 978-1-68440-305-9[Print]; ISBN 978-1-68440-306-6[Electronic]), Clinical and Laboratory Standards Institute;USA; 2025.; https://clsi.org/shop/standards/m100/

[32] Zwir I, Latifi T, Perez JC et al. The promoter architectural landscape of the Salmonella PhoP regulon. Mol Microbiol. 2012;84:463–85. 10.1111/j.1365-2958.2012.08036.x 22435712 PMC3335776

[33] Harari O, del Val C, Romero-Zaliz R et al. Identifying promoter features of co-regulated genes with similar network motifs. 2009;10:S1. 10.1186/1471-2105-10-S4-S1 PMC2681069 19426448

[34] Tapia-Pastrana G, Chavez-Duenas L, Lanz-Mendoza H et al. VirK is a periplasmic protein required for efficient secretion of plasmid-encoded toxin from enteroaggregative *Escherichia coli*. Infect Immun. 2012;80:2276–85. 10.1128/IAI.00167-12 22547550 PMC3416465

[35] Brodsky IE, Ernst RK, Miller SI et al. *mig-14* is a *Salmonella* gene that plays a role in bacterial resistance to antimicrobial peptides. J Bacteriol. 2002;184:3203–13. 10.1128/JB.184.12.3203-3213.2002 12029036 PMC135090

[36] Li LF, Xu XX, Cheng P et al. *Klebsiella pneumoniae* derived outer membrane vesicles mediated bacterial virulence, antibiotic resistance, host immune responses and clinical applications. Virulence. 2025;16:2449722. 10.1080/21505594.2025.2449722 39792030 PMC11730361

[37] Lin J, Huang S, Zhang Q. Outer membrane proteins: key players for bacterial adaptation in host niches. Microbes Infect. 2002;4:325–31. 10.1016/S1286-4579(02)01545-9 11909743

[38] Hussein RA, Al-Kubaisy SH, Al-Ouqaili MTS. The influence of efflux pump, outer membrane permeability and β-lactamase production on the resistance profile of multi, extensively and pandrug resistant *Klebsiella pneumoniae*. Journal of Infection and Public Health. 2024;17:102544. 10.1016/j.jiph.2024.102544 39321604

[39] Prasad SV, Fiedoruk K, Zakrzewska M et al. Glyoxylate shunt and pyruvate-to-acetoin shift are specific stress responses induced by colistin and ceragenin CSA-13 in *Enterobacter hormaechei* ST89. Microbiology. 2023;11:e01215–23. 10.1128/spectrum.01215-23 PMC10434160 37338344

[40] Anandan A, Vrielink A. Structure and function of lipid A–modifying enzymes. Ann NY Acad Sci. 2019;1459:19–37. 10.1111/nyas.14244 31553069

[41] Bhushan G, Castano V, Wong Fok Lung T et al. Lipid A modification of colistin-resistant *Klebsiella pneumoniae* does not alter innate immune response in a mouse model of pneumonia. Infect Immun. 2024;92:e0001624. 10.1128/iai.00016-24 38771050 PMC11237409

[42] Needham BD, Trent MS. Fortifying the barrier: the impact of lipid A remodelling on bacterial pathogenesis. Nat Rev Micro. 2013;11:467–81. 10.1038/nrmicro3047 PMC6913092 23748343

[43] Yang Q, Li M, Spiller OB et al. Balancing *mcr-1* expression and bacterial survival is a delicate equilibrium between essential cellular defence mechanisms. Nat Commun. 2017;8:2054. 10.1038/s41467-017-02149-0 29233990 PMC5727292

[44] Li L, Ma J, Cheng P et al. Roles of two-component regulatory systems in *Klebsiella pneumoniae*: regulation of virulence, antibiotic resistance, and stress responses. Microbiol Res. 2023;272:127374. 10.1016/j.micres.2023.127374 37031567

[45] Mlynarcik P, Kolar M. Molecular mechanisms of polymyxin resistance and detection of *mcr* genes. Biomed Pap Med Fac Univ Palacky Olomouc Czech Repub. 2019;163:28–38. 10.5507/bp.2018.070 30439931

[46] Wang Y, Luo Q, Xiao T et al. Impact of polymyxin resistance on virulence and fitness among clinically important gram-negative bacteria. Engineering. 2021;13:178–85. 10.1016/j.eng.2020.11.005

[47] Wand ME, Bock LJ, Sutton JM. Retention of virulence following colistin adaptation in *Klebsiella pneumoniae* is strain-dependent rather than associated with specific mutations. J Med Microbiol. 2017;66:959–64. 10.1099/jmm.0.000530 28741998

[48] Choi M-J, Ko KS. Loss of hypermucoviscosity and increased fitness cost in colistin-resistant *Klebsiella pneumoniae* sequence type 23 strains. Antimicrob Agents Chemother. 2015;59:6763–73. 10.1128/AAC.00952-15 26282408 PMC4604379

