# Supplementary material for: PhoP-regulated VirK acts as an accessory factor to maintain virulence in polymyxin-resistant Klebsiella pneumoniae
Source: Nucleic Acids Res. 2026 Apr 25;54(8):gkag290. doi: 10.1093/nar/gkag290 (PMC13109725; doi:10.1093/nar/gkag290)
Supplement: gkag290_Supplemental_Files [file gkag290_supplemental_files.zip › Figure S.pdf]

Attachment

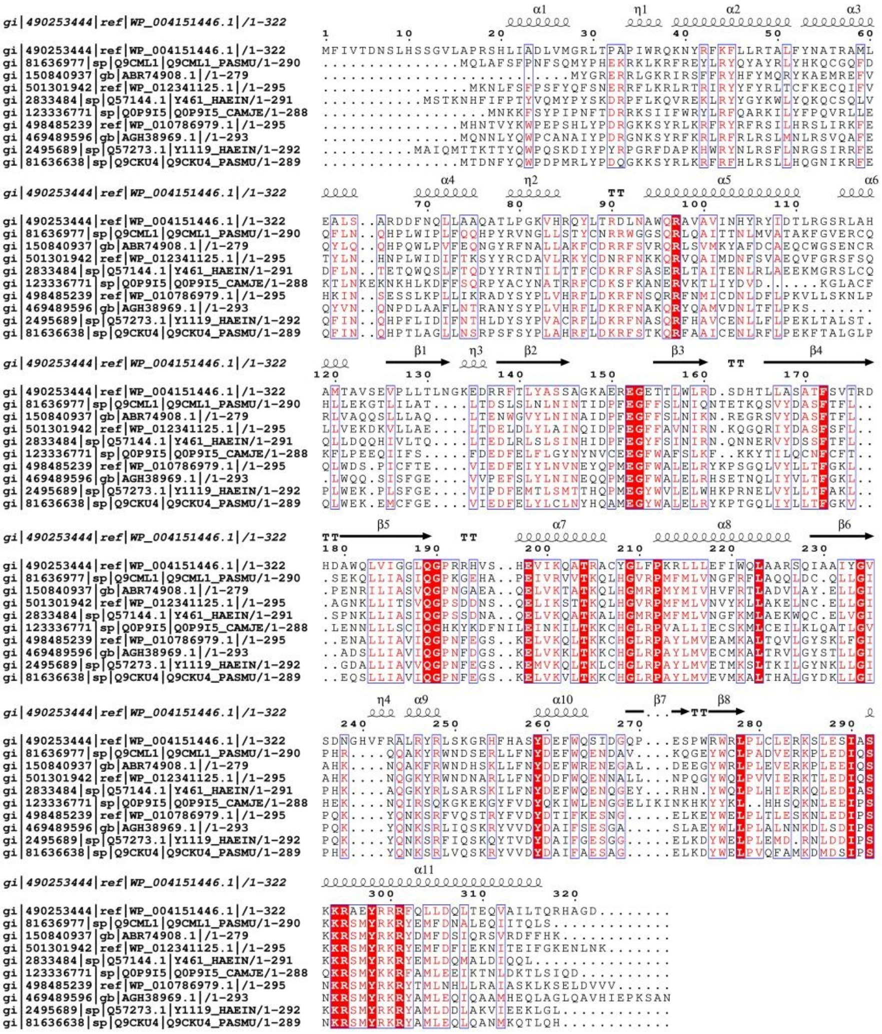

Fig.S1 | The VirK homologues with other VirK/YbjX family by GenBank database. The results were visualized with ESPript 3.0.

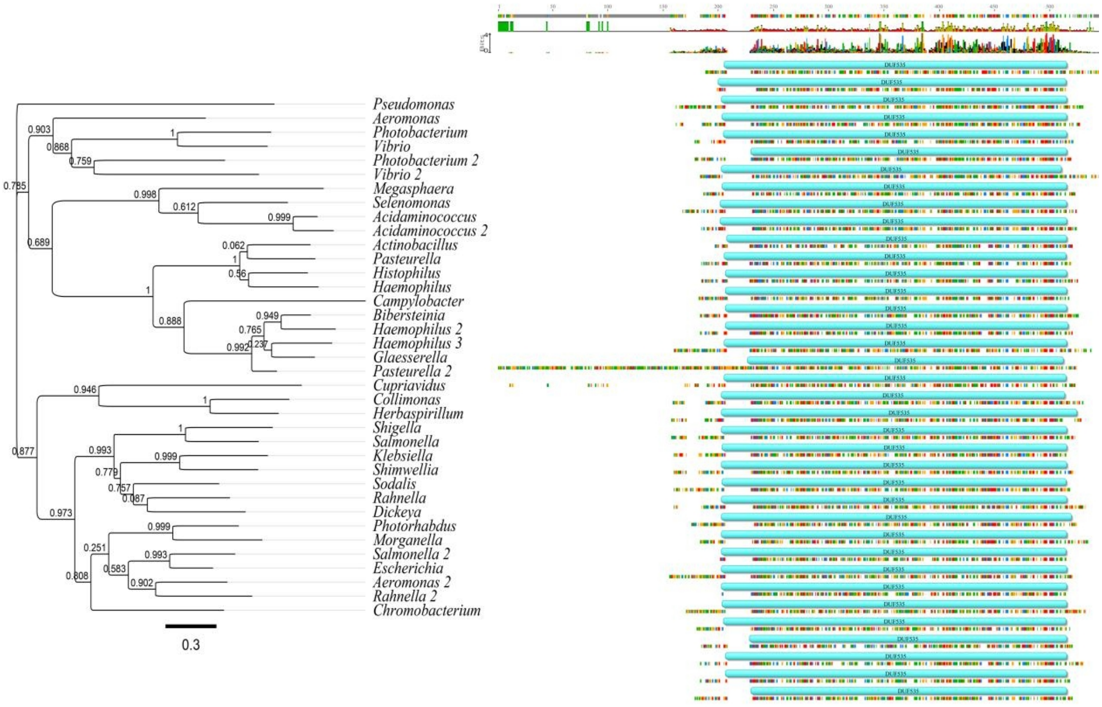

**Fig.S2 | **VirK homologues in VirK/YbjX family possess the same conserved amino acid sequence at the α-helixes.

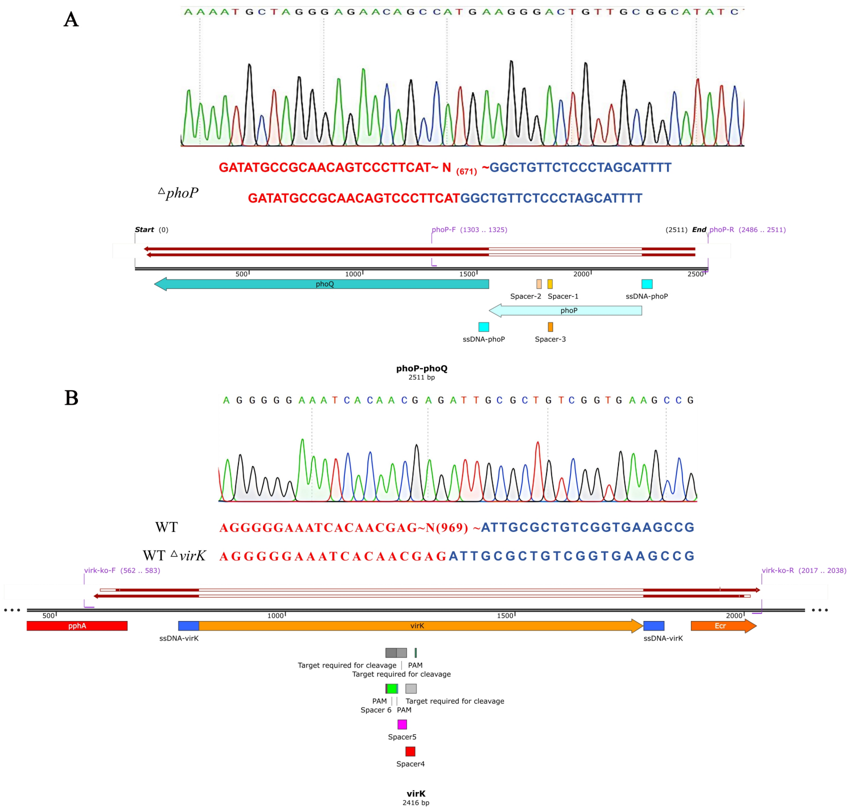

Fig.S3 | a Knockout of the *phoP* in Mut-S by CRISPR-Cas9 system. **b** Knockout of the *virK* in Kpn2146 by CRISPR-Cas9 system.

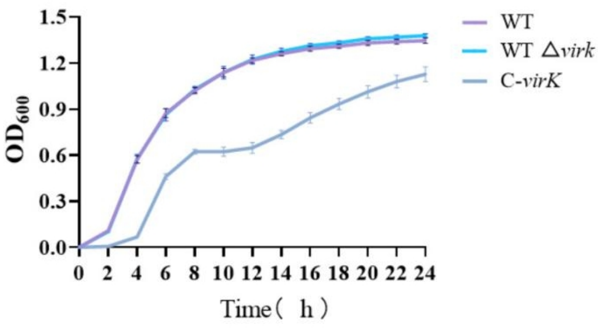

Fig.S4 | Growth curves of the Kpn2146 with or without *virK* gene using the Bioscreen-C automated growth curve analysis system.

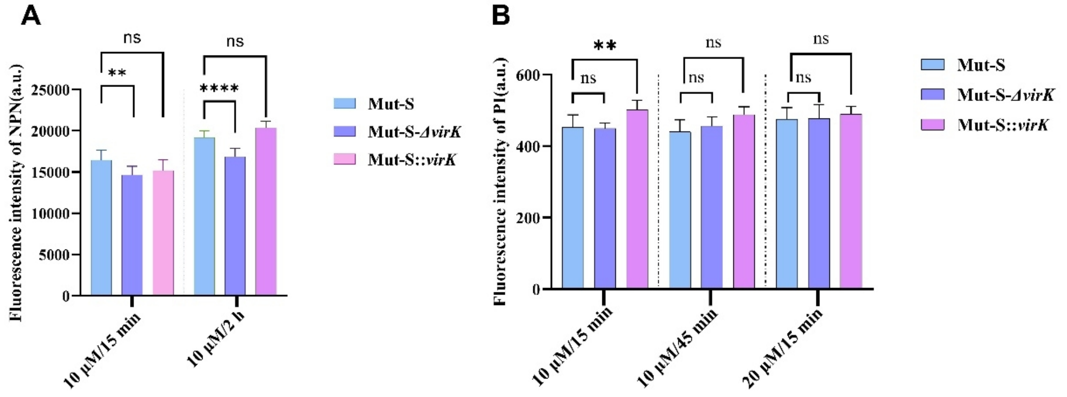

Fig.S5 | NPN and PI uptake experiments to determine the membrane permeability of different strains. a 10 μM NPN treatment for 15 minutes and 2 hours. b 10 μM PI treatment for 15 minutes and 45 minutes, or 20 μM PI treatment for 45 minutes. The fluorescence intensity reflects the internal flow rate of NPN and PI, and the higher the value is, the stronger the membrane permeability and membrane damage. The data are presented as the mean ± SD (n=9), with *p* values adjusted for multiple comparisons determined using one-way ANOVA. a.u., arbitrary units; ns, no statistically significant difference; **, p<0.01,****, p<0.0001
