# Supplementary material for: PhoP-regulated VirK acts as an accessory factor to maintain virulence in polymyxin-resistant Klebsiella pneumoniae
Source: Nucleic Acids Res. 2026 Apr 25;54(8):gkag290. doi: 10.1093/nar/gkag290 (PMC13109725; doi:10.1093/nar/gkag290)
Supplement: gkag290_Supplemental_Files [file gkag290_supplemental_files.zip › Table S.pdf]

Table S1. Strains and plasmids used in this study

Strains	Description	Reference
Kpn*2146*	WT	Lab stock
Mut-S	polymyxin S_2-resistant Kpn*2146*	[1]
Mut-S-Δ*phoP*	*phoP* gene knockout	This study
Mut-S-C-Δ*phoP*	*phoP* gene complementation	This study
Mut-S-C-*mgrB*	*mgrB* gene complementation	This study
BL21(DE3)-6×His-*phoP*	His-PhoP protein expression in BL21(DE3)	This study
Kpn*2146*-Flag-*phoP*	Flag-PhoP protein expression in Kpn*2146*	This study
Kpn*2146*-Δ*virK*	*virK* gene knockout	This study
Kpn*2146*-C-Δ*virK*	*virK* gene complementation	This study
Mut-S-Δ*virK*	*virK* gene knockout	This study
Mut-S-C-ΔvirK	*virK* gene complementation	This study
Kpn*2146*-C-Δ*virK-his6*	*virK* gene complementation with 6×His tag	This study
E. coli top10	Used for efficient plasmid DNA cloning and ensuring stable replication of high copy plasmids	This study
**Plasmids**	**Description**	**Reference**
pCasKP-apr	Expresses Cas9 proteins in *K. pneumoniae* (Apr^r)	Lab stock
pSGKP-spe	Plasmid expressing sgRNA in *K. pneumoniae* (Spe^r)	Lab stock
pSGKP-spe_*phoP*_1	pSGKP-spe derivative with *phoP* spacer 1	This study
pSGKP-spe_*phoP*_2	pSGKP-spe derivative with *phoP* spacer 2	This study
pSGKP-spe_*phoP*_3	pSGKP-spe derivative with *phoP* spacer 3	This study
pSGKP-spe_*virK*_1	pSGKP-spe derivative with *virK* spacer 1	This study
pSGKP-spe_*virK*_2	pSGKP-spe derivative with *virK* spacer 2	This study
pSGKP-spe_*virK*_3	pSGKP-spe derivative with *virK* spacer 3	This study
pTOPO-*mgrB* (Apr^r)		[1]
pTOPO-*phoP* (Apr^r)		This study
pTOPO-flag-*phoP* (Apr^r)		This study
pEASY-*virK* (Apr^r)		This study
pEASY-*virK-his6* (Apr^r)		This study
pET28a(Kan^r)	Low-copy-no. plasmid (Kan^r)	Lab stock
pET28a-6×His-*phoP*	pET28a(Kan^r) derivative with 6×his-*phoP*	This study
pET28a-*virK-his6*	pET28a(Kan^r) derivative with *virK-his6*	This study

Table S2. Primers used in this study

Description	Primers	Sequence (5' to 3')
RT-qPCR	*mgrB*-RT-F[2]	GTTGCTGTGGACTCAGATGCTT
	mgrB-RT-R[2]	TGCCGCTGAAAAACTGAACA
	phoP-RT-F[3]	TCGATGCGGCGGAAGAT
	phoP-RT-R[3]	GCGATATCCGGGAGATGTTC
	23S-44F[2]	AGGCGATGAAGGACGTGCTA
	23S-119R[2]	TTCGGACATCGCCGGTTATA
ChIP-qPCR	*1662virK*-1F	ACGGCAAATATAGTCCGCCA
	1662virK-1R	GCAGGCTATTGTCTGTCACG
	1662virK-2F	ATTCCAGCAAAGGTCCACGG
	1662virK-2R	CCTCGTTGTGATTTCCCCCT
	1662virK-3F	GATATTACGCCAGTCGCTGC
	1662virK-3R	TGGCGGACTATATTTGCCGT
	1662phoQ-1F	CTTTGCTGACCACTTTGCCG
	1662phoQ-1R	GAGCTGTCGGTGAATGACCA
	1662phoQ-2F	TGGTGACGTAATCATCCGCC
	1662phoQ-2R	TTATCACTGATCCGCCGCTG
	1662phoQ-3F	AAAGCTGGAGCATCAGCGAA
	1662phoQ-3R	GATCAAGCTGACCGCCTTTG
Chip-Seq	*phoP-F-F*	ATGGATTACAAGGACGACGATGACAAGATGCGCGT
	phoP-F-R	TCAGCGCAATTCGAACAGATAG
	pTOPO-P-F-F	CGAGTACGCGCATCTTGTCATCGTCGTCCTTGTAAT
	pTOPO-P-F-R	CACGGTGCGCGGCCAGGGCTATCTGTTCGAATTGC
EMSA	E-*virk*-F	GCATCGCCCTTGACGTACCAG
	E-*virk*-R	CTCGTTGTGATTTCCCCCTAT
	E-*mgrB*-F	TTACCACGGAATAAATTTATTAATAGTGC
	E-*mgrB*-R	CCTCACTGTGGAATAACACCCCA
	E-16S rRNA-F	CGTTACCCGCAGAAGAAGCACCG
	E-16S rRNA-R	CGCCTTCGCCACCGGTATTCCTC

Table S3. Primers used for *phoP* knockdown in this study.

phoP-ko	Spacer1-F（*phoP*）	TAGT CGGGAGAGGTCGACCTGGAA
	Spacer1-R（*phoP*）	AAAC TTCCAGGTCGACCTCTCCCG
	Spacer2-F（*phoP*）	TAGT TCAAAGGCGGTCAGCTTGAT
	Spacer2-R（*phoP*）	AAAC ATCAAGCTGACCGCCTTTGA
	Spacer3-F（*phoP*）	TAGT GAGAGGTCGACCTGGAACGG
	Spacer3-R（*phoP*）	AAAC CCGTTCCAGGTCGACCTCTC
	phoP-ko-F	TCGGGCTTTCCATATTCAGGTTT
	phoP-ko-R	GACATAATGGATAACCTGCGACGTTC
	ssDNA-*phoP*-1	AACCCGTAATGACAGCGGGAAGATATGCCGCAACAGTCCCTTCATGGCTGTTCTCCCTAGCATTTTCGATAGTAGTCTGACGCTCATTAT
	ssDNA-*phoP*-2	ATAATGAGCGTCAGACTACTATCGAAAATGCTAGGGAGAACAGCCATGAAGGGACTGTTGCGGCATATCTTCCCGCTGTCATTACGGGTT
***phoP* gene complementation**	*phoP*-C-F	ATTTCACACAGGAAACAGCTATGCGCGTACTCGTGGTTG
	phoP-C-R	TATCTGCAGAATTCGCCCTTTCAGCGCAATTCGAACAGATAGC
	pTOPO-P-F	TCAACCACGAGTACGCGCATAGCTGTTTCCTGTGTGAAATTGT
	pTOPO-P-R	ATCTGTTCGAATTGCGCTGAAAGGGCGAATTC

Table S4. Primers used for *virk* knockdown in this study.

***virK*-ko**	Spacer4-F	TAGTGTGAGGGTGAAACCACTCTG
	Spacer4-R	AAACCAGAGTGGTTTCACCCTCAC
	Spacer5-F	TAGTACGTTCGGCTTTGCCTGCCG
	Spacer5-R	AAACCGGCAGGCAAAGCCGAACGT
	Spacer6-F	TAGTAGGCATAAAGAGTAAACCGG
	Spacer6-R	AAACCCGGTTTACTCTTTATGCCT
	virk-ko-F	GACGCAGGCGATTCATCAGCAG
	virk-ko-R	GCGGAAAATATTATTGCGGAAC
	ssDNA-*virk*-1	AGCTTGTCAGTGGCTCGCTTTAAAATAGGGGGAAATCA CAACGAGATTGCGCTGTCGGTGAAGCCGAATTACAACA TGGCGTTTGCTTAT
	ssDNA-*virk*-2	ATAAGCAAACGCCATGTTGTAATTCGGCTTCACCGACA GCGCAATCTCGTTGTGATTTCCCCCTATTTTAAAGCGAG CCACTGACAAGCT
***virk* gene complement ation**	*virK*-C-F	TCACACAGGAAACAGCTGTGTTTATCGTGACAGACAAT AGCCTG
	virK-C-R	CCACACCCGCCGCGCTTAGTCACCGGCATGCCGC
	pEASY-V-F	GCGCGGCGGGTGTGGTGGTTACGCG
	pEASY-V-R	AGCTGTTTCCTGTGTGAAATTGTTA
	virK-H-F	TCACACAGGAAACAGCTGTGTTTATCGTGACAGACAAT AGCCTG
	virK-H-R	CCACACCCGCCGCGCTCAGTGGTGGTGGTGGTGGTGC
	pEASY-V-H-F	GCGCGGCGGGTGTGGTGGTTACGCG
	pEASY-V-H-R	AGCTGTTTCCTGTGTGAAATTGTTA

Table S5. Probe for DNase I footprinting assay.

**Probe Sequence**	CAGATATTACGCCAGTCGCTGCCGTTAATACGTTGATACATATAATTACCTC TCAGCGGTAATTATAGGTAATTCCAGCAAAGGTCCACGGAATTTAATTTAC ACCTCAATCAATTTTAAACAGACGGCAAATATAGTCCGCCAGAGGATTAAG CTTGTCAGTGGCTCGCTTTAAAATAGGGGGAAATCACAACGAGGTGTTTAT CGTGACAGACAATAGCCTGCATTCATCCGGCGTGCTAGCGCCGCGCTCGCA TCTTATTGCCGATCTGGTGATGGGCAGATTGACACCTGCCCCCATCTGGCGG CAAAAAAATTACCGCTTTAAG

Table S6. Clinical strain information

sequencing_id	organism	Surveillance	specimen_type	specimen_source	gender	age	country	province	COL	KPC	MLST
C1167	Klebsiella pneumoniae	CRE network	blood	Homo sapiens	male	63	China	Huibei	0.25	KPC-2	ST11
C1218	Klebsiella pneumoniae	CRE network	sputum	Homo sapiens	male	44	China	Henan	0.25	KPC-2	ST11
C1225	Klebsiella pneumoniae	CRE network	sputum	Homo sapiens	male	72	China	Henan	0.25	KPC-2	ST11
C1227	Klebsiella pneumoniae	CRE network	sputum	Homo sapiens	male	92	China	Henan	0.25	KPC-2	ST11
C1250	Klebsiella pneumoniae	CRE network	urine	Homo sapiens	male	87	China	Fujian	0.25	KPC-2	ST11
C1260	Klebsiella pneumoniae	CRE network	sputum	Homo sapiens	male	49	China	Henan	0.25	KPC-2	ST11
C1261	Klebsiella pneumoniae	CRE network	blood	Homo sapiens	male	90	China	Henan	0.25	KPC-2	ST11
C1262	Klebsiella pneumoniae	CRE network	blood	Homo sapiens	male	78	China	Henan	0.25	KPC-2	ST11
C677	Klebsiella pneumoniae	CRE network	blood	Homo sapiens	male	63	China	Guangdong	0.25	KPC-2	ST11
C1459	Klebsiella pneumoniae	CRE network	urine	Homo sapiens	female	93	China	Beijing	32	KPC-2	ST11
C2582	Klebsiella pneumoniae	CRE network	blood	Homo sapiens	female	4m	China	Shandong	8	KPC-2	ST11
C6609	Klebsiella	CRE network	serum	Homo sapiens	male	80	China	Shandong	>8	KPC-2	ST11

	pneumoniae										
C7289	Klebsiella pneumoniae	CRE network	NA	Homo sapiens	NA	NA	China	Fujian	>8	KPC-2	ST11
C7367	Klebsiella pneumoniae	CRE network	urine	Homo sapiens	female	NA	China	Beijing	>8	KPC-2	ST11
C7579	Klebsiella pneumoniae	CRE network	blood	Homo sapiens	male	74	China	Henan	>8	KPC-2	ST11
C7864	Klebsiella pneumoniae	CRE network	urine	Homo sapiens	male	3m	China	Hebei	>8	KPC-2	ST11
C7783	Klebsiella pneumoniae	CRE network	NA	Homo sapiens	NA	NA	China	Guagndong	>8	KPC-2	ST11
C7784	Klebsiella pneumoniae	CRE network	NA	Homo sapiens	NA	NA	China	Guangdong	>8	KPC-2	ST11

1. Li, H.B., Sun, L., Qiao, H. et al.. Polymyxin resistance caused by large-scale genomic inversion due to IS*26* intramolecular translocation in *Klebsiella pneumoniae*. *Acta Pharm Sin B*. 2023; 13: 3678–93.
2. Cheng, Y.H., Lin, T.L., Pan, Y.J. et al.. Colistin resistance mechanisms in *Klebsiella pneumoniae* strains from Taiwan. *Antimicrob Agents Chemother*. 2015; 59: 2909–13.
3. Cheng, Y.H., Lin, T.L., Lin, Y.T. et al.. Amino acid substitutions of CrrB responsible for resistance to colistin through CrrC in *Klebsiella pneumoniae*. *Antimicrob Agents Chemother*. 2016; 60: 3709–16.
